# Identification and validation of diagnostic markers of atherosclerosis progression via bioinformatics strategies

**DOI:** 10.1371/journal.pone.0336139

**Published:** 2025-12-05

**Authors:** Yun Mao, Yang Xiao, Xia Hu

**Affiliations:** 1 Department of Cardiology, The First Afﬁliated Hospital of Zhengzhou University, Zhengzhou, People's Republic of China; 2 Centre for Cardiovascular Diseases, Henan Key Laboratory of Hereditary Cardiovascular Diseases, Zhengzhou, People's Republic of China; 3 Department of Critical Care Medicine, The First Affiliated Hospital of Zhengzhou University, Zhengzhou, Henan, China; Dasman Diabetes Institute, KUWAIT

## Abstract

**Background:**

Arteriosclerosis (AS) is a leading cause of cardiovascular disease, imposing significant burdens on families and society. However, the underlying mechanisms remain unclear. This study aims to elucidate these mechanisms and explore potential pharmacological treatments through the integration of bioinformatics.

**Methods:**

The GSE28829 dataset was retrieved from the GEO database. Differential gene expression between early- and late-stage AS plaques in GSE28829 was identified via the limma package. We focused on the intersecting genes associated with endoplasmic reticulum stress and mitochondrial damage. LASSO regression analysis was applied to pinpoint potential core genes associated with AS. An protein interaction network was constructed, with a focus on the hub genes within this network. Assessment of immune cell infiltration levels was achieved via the ssGSEA method and confirmed via CIBERSORT. We used the DrugBank database to predict small molecule drugs that could intervene in AS progression.

**Results:**

In this study, DEGs associated with AS were identified, and hub genes, including *FCGR3A, ITGB2, TYROBP, FCGR2B, CTSS, FCER1G, CD86, TLR2, C1QB, and C1QA*, were identified. We also found a strong correlation between the hub genes and immune processes, indicating that ER stress and mitochondrial damage were correlated with the activation of immune processes. Additionally, a diagnostic model for AS was established, demonstrating the substantial predictive value of these hub genes.

**Conclusions:**

This study reveals the involvement of genes related to endoplasmic reticulum stress and mitochondrial damage in the pathogenesis of AS. These hub genes offer new directions for further research and the development of pharmacological treatments.

## 1. Introduction

Atherosclerosis (AS) has become a significant contributor to cardiovascular diseases, leading to severe consequences such as infarction and necrosis [1]. The mechanisms underlying AS are complex and not fully understood, likely involving multiple physiological processes and factors [2].

Studies have identified endoplasmic reticulum (ER) stress and mitochondrial damage as key factors in the development of AS [3]. Disruptions in ER function (e.g., oxidative stress, inflammation, or lipid overload) can induce ER stress, leading to the activation of the unfolded protein response (UPR). However, excessive ER stress and the UPR trigger inflammatory responses and apoptosis, significantly impacting the progression of AS. Additionally, ER stress can disrupt the function of AS-related cells (such as endothelial cells, smooth muscle cells, and macrophages) [3]. Mitochondrial damage in AS can increase oxidative stress, harm vascular endothelial cells, and promote foam cell formation and inflammation. This damage might also increase inflammatory cell recruitment and activation, exacerbating vascular inflammation and plaque instability. The interplay between ER stress and mitochondrial damage might synergistically drive the progression of AS [4,5].

In this study, we analyzed the GSE28829 dataset from the GEO database, focusing on genes related to ER stress and mitochondrial damage. We identified 72 DEGs and further selected 7 core genes to construct a diagnostic model for AS. Our study also identified potential core genes for AS, constructed a protein interaction network, and selected 10 hub genes. We further validated drugs related to these genes. Overall, these genes elucidate the mechanisms involved in the development of AS, providing a foundation for future diagnostics and treatment.

## 2. Materials and methods

**Fig 1:**
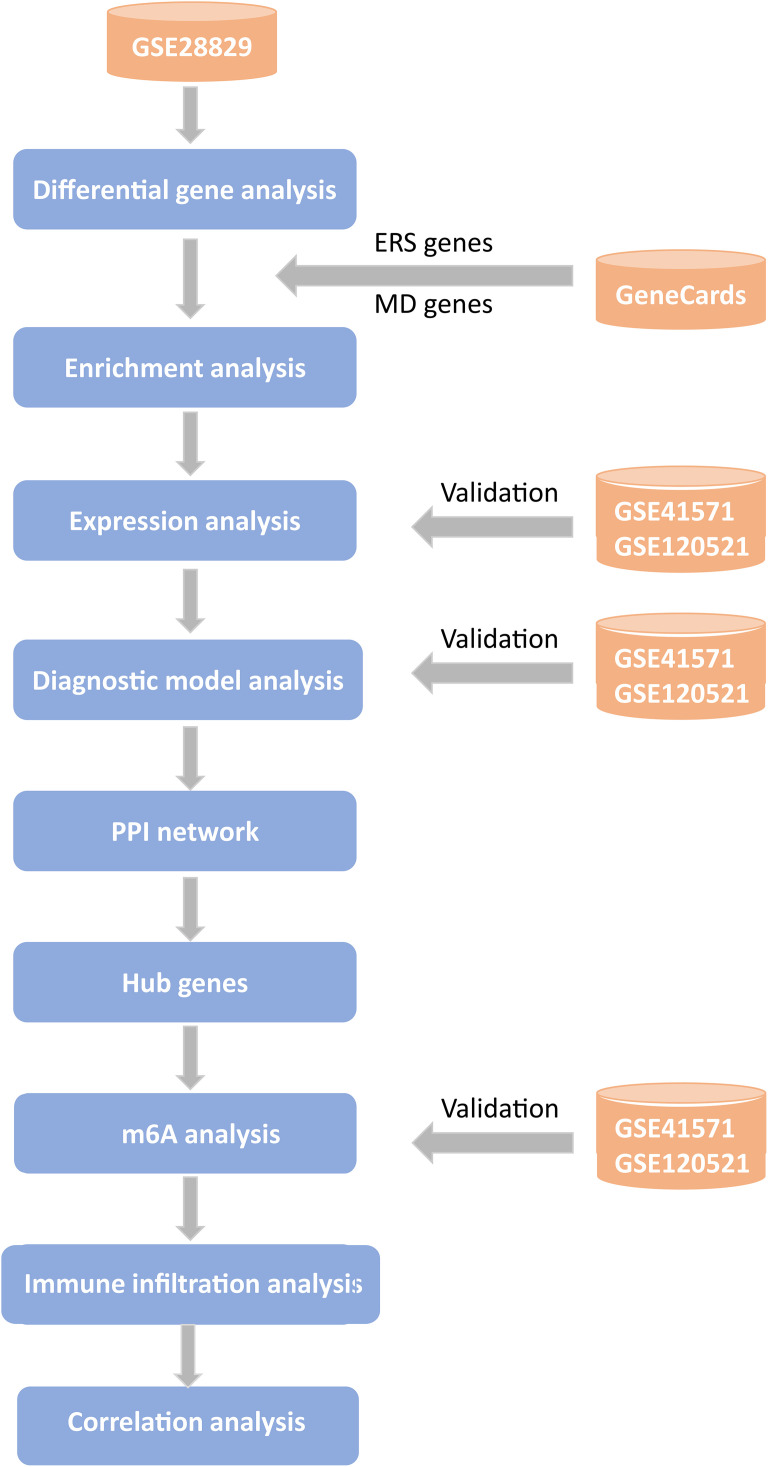
Workflow. The ERS gene represents genes associated with ER stress; the MD genes represent genes associated with mitochondrial damage. The PPI network represents the protein‒protein interaction network.

The study was designed and performed following the framework in Fig 1.

### 2.1. Data and preprocessing

The GSE28829 dataset from human AS patients was downloaded from the GEO database [6]. This dataset contained a total of 29 patients, 13 in early-stage and 16 in advanced-stage. Microarray sequencing data in raw CEL format were read using the R package affy (v1.70.0), and the background correction was performed using the RMA method [7]. Standardized and nonstandardized were processed separately to compare distribution differences, then visualized using box plots. Principal component analysis (Principal Component Analysis, PCA) was performed using the R package FactoMineR (v2.4) and visualized by scatter plot [8]. In addition, to further validate the conclusions of subsequent studies, two other datasets from the GEO database, GSE41571 (https://www.ncbi.nlm.nih.gov/geo/query/acc.cgi?acc=GSE41571) and GSE 120521 (https://ww.ncbi.nlm.nih.gov/geo/query/acc.cgi?acc=GSE120521) were collected as a validation set [9]. The GSE41571 dataset contains 11 patients, including 5 unstable plaques and 6 with stable plaques. The GSE 120521 dataset contains 4 unstable plaques and 4 stable plaques. The batch effects were removed using Harmony.

### 2.2. Differential gene expression analysis

To compare the differences in gene expression between early and advanced AS patients, we performed differential expression analysis via the R package limma (v3.48.3) [10]. |log2-fold change| (|log2FC|) ≥=1 and P value <0.05 were set as the thresholds. Genes with log2FC > 1 and the P value <0.05 were considered upregulated genes, whereas genes with log2FC < −1 and a P value <0.05 were considered downregulated genes. Volcano maps and heatmaps were used to visualize significant DEGs, and volcano plots were drawn via the R package ggplot2 (v3.3.5) [11]. Heatmaps were plotted via the R package pheatmap (v1.0.12) [12–14].

### 2.3. Gene enrichment analysis

To further reveal the biological functions affected by DEGs related to ER stress and mitochondrial damage, we searched the GeneCards database with the key words “endoplasmic reticulum stress” and “mitochondrial damage”, analyzed the intersecting genes, and then performed gene enrichment analysis for the intersecting genes [15].

Gene ontology (GO; http://geneontology.org/) enrichment analysis is a common method used to study the large-scale functional enrichment of genes in different dimensions and at different levels. KEGG is generally conducted at three levels: biological process (BP), molecular function (MF) and cellular component (CC) [16]. KEGG (Kyoto Encyclopedia of Genes and Genomes, https://www.genome.jp/kegg/) is a widely used database that stores information about genomes, biological pathways, diseases, and drugs [17]. GO functional annotation and KEGG biological pathway enrichment analysis revealed significantly enriched biological processes and pathways, the enrichment results were visualized via the R package GO plot (v1.0.2), and the significance threshold for the enrichment analysis was set to P < 0.05 [13].

Gene set enrichment analysis (GSEA) is a computational method used to determine whether a set of predefined sets of genes shows significant differences between two biological states and is usually used to estimate changes in pathway and biological process activity in expression dataset samples [14]. To investigate the differences in biological processes between the two patient groups, we downloaded the reference gene set “c2.cp.kegg” from the gene expression profile dataset. v7.4. entrez.gmt” from the MSigDB database (https://www.gsea-msigdb.org/gsea/msigdb/). We also focused on the cGAS-STING, STING-IRF3, and STING-NLRP3 pathways, which are considered to be associated with ER stress damage. We performed enrichment analysis and visualization of the dataset via the GSEA method in the R package clusterProfiler (v4.0.5) [18]. A P value <0.05 was considered to indicate statistical significance.

### 2.4. Expression analysis of genes associated with ER stress and mitochondrial damage

To analyze the differences and correlations of genes in early and advanced AS patients, the transcriptomic expression profiles of DEGs related to ER stress and mitochondrial damage were analyzed. Group box plots were plotted via the R packages ggplot 2 (v3.3.5) and ggpubr (v0.4.0) [11]. Differences were analyzed via the Wilcoxon test, and a P value <0.05 was considered to indicate statistical significance. The correlation plots were drawn via the R package corrplot (v0.90).

### 2.5. Construction of the diagnostic model

Owing to the important effects of ER stress and the mitochondrial damage process, patients may have different levels of cellular ER stress and mitochondrial damage, so it is extremely feasible to construct diagnostic models based on cellular ER stress and mitochondrial damage-related genes.

Here, we first screened all cellular genes via LASSO regression for DEGs associated with ER stress and mitochondrial damage via the R package glmnet (v4.1-2) to implement the method and select the best lambda value. Only genes whose coefficient was not zero were retained after regression, and these genes were used to construct a diagnostic model. The corresponding coefficients were presented as forest plots via the R package forestplot (v2.0.1) [19]. To validate the predictive efficacy of the diagnostic model, the area under the ROC curve (AUC) was subsequently calculated via the R package pROC (v1.18.0) [19]. Finally, we confirmed the robustness of the model via the external datasets GSE41571 and GSE120521. The area under the ROC curve (AUC) was calculated to assess the predictive efficacy of the model.

### 2.6. Construction of a protein interaction network of genes involved in ER stress and mitochondrial damage

The differentially expressed genes related to ER stress and mitochondrial damage were used to construct a protein‒protein interaction (PPI) network. The DEGs were used as inputs in the STRING database, and the confidence threshold was set as 0.4. The PPIs were further analyzed via Cytoscape (v3.8.2) software [20]. The MCC algorithm (cytoHubba (v0.1)) was used to mine hub nodes, and we took the top 10 nodes as hub nodes, which have high levels of connection with other nodes [21]. Then, we analyzed the hub nodes, including the miRNAs and transcription factors of the hub nodes, via the miRNet (https://www.mirnet.ca) database [22]. In addition, we predicted the small molecule drugs bound to the hub node in the Drug Bank (https://go.drugbank.com/drugs). The predicted results were processed and visualized via Cytoscape (v3.8.2) [20,23].

### 2.7. Analysis of immune infiltration

The immune microenvironment is mainly composed of immune cells, inflammatory cells, fibroblasts, stromal tissues, and various cytokines and chemokines. The infiltration analysis of immune cells in tissues has an important guiding role in disease research and the prediction of treatment prognosis.

To further explore the relationships between the hub genes and immune cell infiltration levels, all the samples were divided into high- and low-expression groups according to the means of the expression values of the 10 hub genes. Assessment of immune cell infiltration levels was achieved via the R package GSVA (v1.40.1) via the ssGSEA method.

We obtained the marker genes of 28 immune cells from the TISIDB (http://cis.hku.hk/TISIDB) [24] and used them as the background gene set for ssGSEA. The infiltration of all immune cells was presented via box plots and heatmaps, and the heatmaps were plotted via the R package pheatmap (v1.0.12) [12]. In addition, to determine the differences in the degree of association of immune cells in different AS states, we graphed the correlations between immune cells in the high- and low-expression groups via the R package corrplot (v0.90).

To confirm the accuracy of the study results, the infiltration level of immune cells was assessed via another method, CIBERSORT [25]. On the basis of the LM22 background gene set provided by the CIBERSORT website (https://cibersort.stanford.edu/), we calculated the content of 22 immune cells in each sample to obtain their infiltration levels separately. The results are presented via box plots and stacked bars drawn via the R package ggplot 2 (v3.3.5) [11].

We observed that immune cells showing significant differences between patients in the high- and low-expression groups might be more closely associated with hub genes. To explore this potential relationship further, we generated plots comparing the levels of immune cell infiltration with the expression values of the hub genes, which showed a considerable difference. These plots were graphed via the R package ggExtra (v 0.9), with a focus on data with a P value < 0.01 [26].

### 2.8. Statistical analysis

All data calculations and statistical analyses were performed in the R language (v4.1.0). Comparisons of two independent sets of variables were analyzed for differences between nonnormally distributed variables via the Wilcoxon rank sum test. All P values were two-sided, and P < 0.05 was considered statistically significant.

## 3. Results

### 3.1. Differentially expressed genes (DEGs)

The dataset GSE28829 from the GEO database contains a total of 29 patients, including 13 early AS patients and 16 advanced AS patients, and 22,836 sequenced genes (Table 1).

**Table 1:** 29 patients were involved in the dataset GSE28829 from the GEO database, including 13 early AS patients and 16 advanced AS patients.

	GSE28829	GSE41571	GSE120521
**Organism**	Homo sapiens	Homo sapiens	Homo sapiens
**Experiment type**	Expression profiling by array	Expression profiling by array	Expression profiling by high throughput sequencing
**Platforms**	GPL570[HG-U133_Plus_2]	GPL570 [HG-U133_Plus_2]	GPL16791[Illumina HiSeq 2500 (Homosapiens)]
**Sample(number)**			–
Advanced	16	–	–
Early	13	–	–
Ruptured Plaque	–	5	–
Stable Plaque	–	6	4
Unstable Plaque	–	–	4

**Fig 2:**
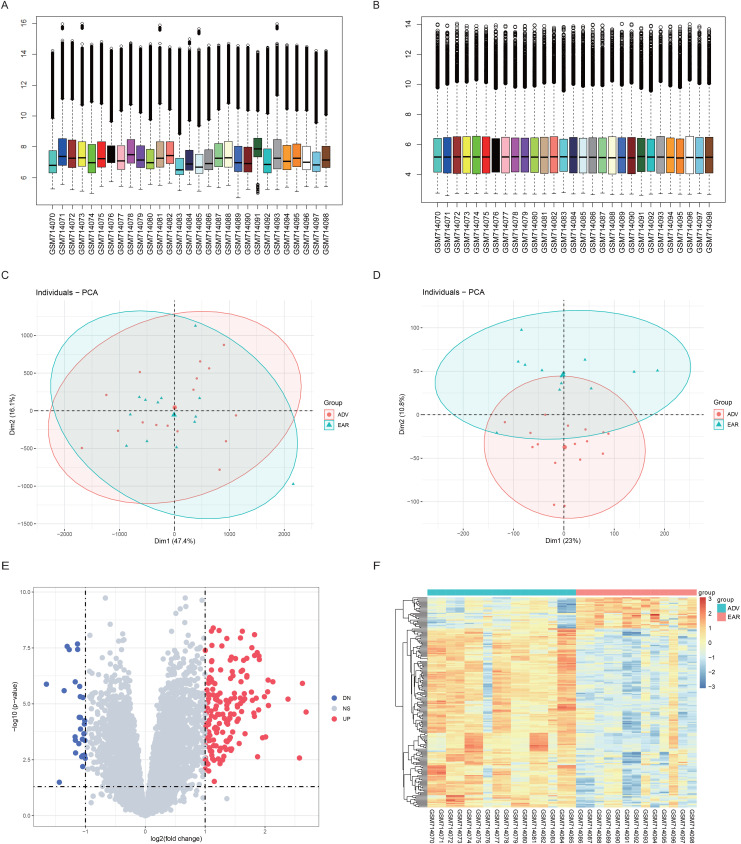
Data preprocessing and differential expression analysis. **A.** Box plot of gene expression before normalization. The x-axis represents the samples, and the y-axis represents the gene expression values. **B.** Boxplots of gene expression after normalization. **C.** PCA dimension reduction diagram before the calibration. The x- and y-axes represent two dimensionality reductions, and the dots in the plot represent samples, with early samples in blue and late samples in red. **D.** PCA dimension reduction map after normalization. **E.** Volcano plots of the DEGs. The x-axis is log2 (fold change), the y-axis is log10 (p value), and each dot represents one gene, with downregulated genes in purple, upregulated genes in red, and black for genes whose expression did not significantly change. **F.** Heatmap of the DEGs. The top color bars represent both groups, with red indicating early expression, green indicating late expression, blue indicating low expression and red indicating high expression.

The raw data were processed via RMA methods, and the data were standardized and nonnormalized separately to determine the differences in the data distributions before and after normalization (Fig 2A-B). The principal component analysis (PCA) method was used to determine whether the gene expression profiles distinguished between patient types (Fig 2C-D). The results revealed that the standardized gene expression profiles had a more uniform distribution between patients after correction for individual patient differences and thus were more favorable for downstream analysis. In addition, the PCA dimensionality reduction results revealed that the standardized gene expression profiles were better able to distinguish between different patient types.

To reveal the biological differences between early-stage and late-stage patients at the transcriptome level, differential gene expression analysis was first conducted with the patients as the group label. After screening by the statistical significance threshold, a total of 189 genes were defined as significant DEGs (Fig 2E-F).

### 3.2. Gene enrichment analysis

To further reveal the biological functions and processes affected by the DEGs related to ER stress and mitochondrial damage, 7157 ER stress-related genes and 9104 mitochondrial damage-related genes were first retrieved from the GeneCards database and then crossed with the DEGs for a total of 72 genes. Then, GO and KEGG enrichment analyses were performed on the basis of the expression profiles of the 72 genes, which were visualized in multiple forms.

**Fig 3:**
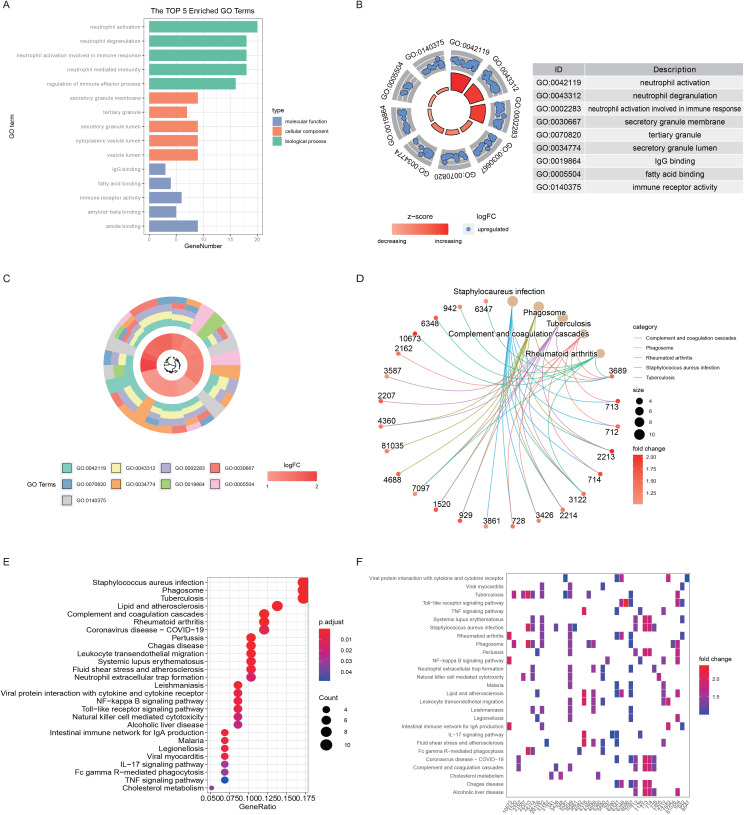
GO/KEGG enrichment analysis of DEGs related to ER stress and mitochondrial damage. **A.** Bar plots of the results of the GO enrichment analysis. The x-axis is the number of genes, and the y-axis is the enriched GO term, which is divided into three colors according to the three levels of BP, CC and MF. Only the top 5 most significant GO terms at each level are shown here. **B.** Circular Fig 1 of the GO enrichment analysis results. The left loop is the GO term ID, corresponding to the right table, and the central loop represents downregulated genes. Each point represents a gene enriched with respect to the GO term. All genes in the graph are upregulated (blue). The inner ring represents the z score, which is closer to the red gene upregulation. The color bar length represents the corrected p value; the longer the color bar represents a smaller p value, the greater the significance. Only the top 3 GO terms of the three levels of BP, CC and MF are shown here. **C.** Circular plot 2 of the results of the GO enrichment analysis. The inner ring in the figure is the gene cluster tree, the middle is the corresponding logFC value of these genes; the closer the red logFC value is, the higher the level of upregulation. The outer ring is the GO terms enriched by these genes. Each GO term uses a color distinction, and only the top 3 BPs and MFs and CCs are shown here. **D.** Spinning plot of the KEGG enrichment results. This graph shows the subordination between genes and pathways. The gene is connected by a string to its corresponding pathway; the left circle represents the gene, the right circle represents the pathway, and the color of the gene represents the logFC value. The closer the red logFC value is, the greater the level of upregulation, and the pathways are distinguished by different colors. **E.** Bubble diagram of the KEGG enrichment results. The x-axis represents the proportion of genes, the total number of genes/DEGs enriched in the pathway, the y-axis represents the pathway name, the dot size represents the number of genes enriched in the pathway, the color indicates the corrected p value, and the smaller the p value is, the closer the red is, and the greater the significance. **F.** Heatmap of the KEGG enrichment results. The X-axis represents the genes, and the y-axis represents the pathways. The colors represent the fold change values of the genes.

The GO enrichment results revealed that the top 5 genes were involved in neutrophil activation, neutrophil degranulation, neutrophil activation involved in the immune response, neutrophil-mediated immunity and the regulation of immune effector processes and were associated mainly with neutrophil-mediated immune processes. This finding suggests a significant correlation between ER stress, mitochondrial damage, and the immune effects of neutrophils. The regulation of immune effector processes might be directly correlated with immunity (Fig 3A-C, Table 2). Importantly, in advanced atherosclerosis, most of the the most significantly enriched GO terms were upregulated compared with those in early atherosclerosis, indicating more active ER stress and mitochondrial damage function in advanced patients. The KEGG enrichment results revealed that the five pathways with the most significant enrichment were *Staphylococcus aureus* infection, the phagosome, tuberculosis, complement and coagulation cascades and rheumatoid arthritis, in which the phagosome was associated with mitochondrial damage and immunity (Fig 3D-F, Table 2).

**Table 2:** GO/KEGG enrichment analysis indicatied a significant correlation between ER stress, mitochondrial damage, and the immune regulation in AS progression.

Description	ONTOLOGY	p.adjust	qvalue
Neutrophil activation	BP	4.02E-12	2.47E-12
Neutrophil degranulation	BP	1.72E-10	1.06E-10
Neutrophil activation involved in immune response	BP	1.72E-10	1.06E-10
Neutrophil mediated immunity	BP	1.88E-10	1.16E-10
Regulation of immune effector process	BP	7.64E-09	4.69E-09
Response to molecule of bacterial origin	BP	1.64E-08	1.01E-08
Response to lipopolysaccharide	BP	8.97E-08	5.51E-08
Microglial cell activation	BP	1.39E-07	8.56E-08
Positive regulation of cytokine production	BP	2.33E-07	1.43E-07
Phagocytosis	BP	4.20E-07	2.58E-07
Macrophage activation	BP	1.04E-06	6.36E-07
Cellular response to molecule of bacterial origin	BP	1.26E-06	7.76E-07
Cellular response to biotic stimulus	BP	3.18E-06	1.95E-06
Humoral immune response	BP	3.18E-06	1.95E-06
Receptor-mediated endocytosis	BP	6.66E-06	4.09E-06
Positive regulation of cell activation	BP	6.77E-06	4.15E-06
Cellular response to lipopolysaccharide	BP	8.00E-06	4.91E-06
Neutrophil chemotaxis	BP	1.56E-05	9.56E-06
Leukocyte cell-cell adhesion	BP	1.56E-05	9.56E-06
Adaptive immune response based on somatic recombination of immune receptors built from immunoglobulin superfamily domains	BP	1.56E-05	9.56E-06
……	……	……	……
Cytokine receptor binding	MF	0.033334	0.024782
Serine-type endopeptidase activity	MF	0.033493	0.0249
G protein-coupled receptor binding	MF	0.042125	0.031317
Carboxylic acid binding	MF	0.043883	0.032624
Phosphatidylcholine binding	MF	0.046282	0.034408
Quaternary ammonium group binding	MF	0.046282	0.034408
KEGG Enrichment analysis results			
Description		p.adjust	qvalue
Staphylococcus aureus infection		1.74E-07	1.29E-07
Phagosome		7.64E-06	5.63E-06
Tuberculosis		2.49E-05	1.83E-05
Complement and coagulation cascades		8.37E-05	6.17E-05
Rheumatoid arthritis		0.000123	9.07E-05
Pertussis		0.000402	0.000297
Chagas disease		0.001841	0.001357
Lipid and atherosclerosis		0.0026	0.001918
Leukocyte transendothelial migration		0.002654	0.001957
Leishmaniasis		0.00331	0.002441
Intestinal immune network for IgA production		0.005005	0.003691
Systemic lupus erythematosus		0.005005	0.003691
Malaria		0.005005	0.003691
Fluid shear stress and atherosclerosis		0.005005	0.003691
Legionellosis		0.006954	0.005129
Viral protein interaction with cytokine and cytokine receptor		0.006954	0.005129
NF-kappa B signaling pathway		0.007083	0.005224
Toll-like receptor signaling pathway		0.007083	0.005224
Viral myocarditis		0.007083	0.005224
Coronavirus disease - COVID-19		0.00984	0.007257
Neutrophil extracellular trap formation		0.016893	0.012459
Natural killer cell mediated cytotoxicity		0.016893	0.012459
Alcoholic liver disease		0.022919	0.016903
IL-17 signaling pathway		0.029284	0.021597
Fc gamma R-mediated phagocytosis		0.031439	0.023187
Cholesterol metabolism		0.032511	0.023977
TNF signaling pathway		0.04821	0.035556

**Fig 4:**
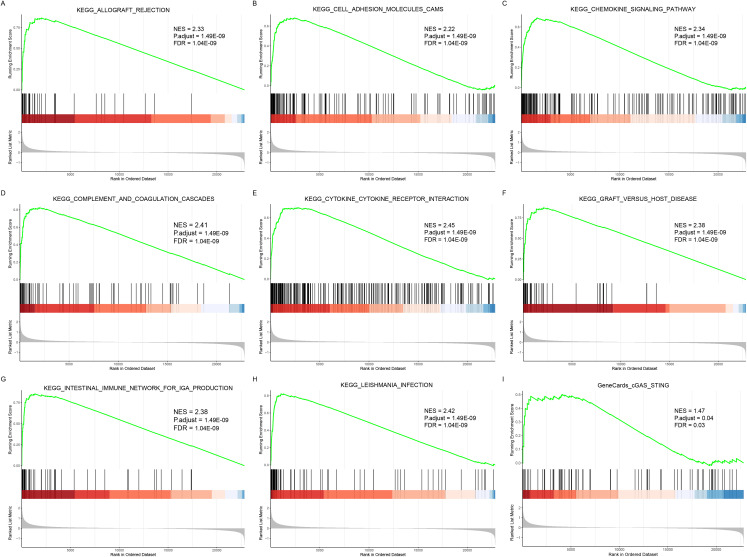
GSEA. A-H sequentially represents the top eight most significant pathways, and I represent the cGAS-STING pathway of particular interest. The x-axis represents the rank of genes in the list of DEGs, the upper y-axis represents the logfc value, with > 0 upregulated genes, < 0 downregulated genes, and the overall left of the curve corresponds to logfc> 0; thus, all these pathways are upregulated pathways.

To further confirm the conclusions of our enrichment analysis, an enrichment analysis was performed for all DEGs based on the KEGG background gene set. The five most significantly enriched pathways were KEGG_ALLOGRAFT_REJECTION, KEGG_CELL_ADHESION_MOLECULES_CAMS, KEGG_CHEMOKINE_SIGNALING_PATHWAY, KEGG_COMPLEMENT_AND_COAGULATION_CASCADES, and KEGG_CYTOKINE_CYTOKINE_RECEPTOR_INTERACTION, and all of them were upregulated pathways that were equally highly enriched with immune-related functions (Fig 4, Table 3). Notably, we also paid special attention to the cGAS-STING, STING-IRF3, and STING-NLRP3 pathways and found that cGAS-STING was significantly upregulated in advanced-stage patients (S5 Fig).

**Table 3:** GSEA Enrichment analysis confirmed all the upregulated pathways were closely related to immune-related functions.

Description	NES	P adjust
KEGG_ALLOGRAFT_REJECTION	2.32997	1.49E-09
KEGG_CELL_ADHESION_MOLECULES_CAMS	2.218522	1.49E-09
KEGG_CHEMOKINE_SIGNALING_PATHWAY	2.338679	1.49E-09
KEGG_COMPLEMENT_AND_COAGULATION_CASCADES	2.413645	1.49E-09
KEGG_CYTOKINE_CYTOKINE_RECEPTOR_INTERACTION	2.449173	1.49E-09
KEGG_GRAFT_VERSUS_HOST_DISEASE	2.381635	1.49E-09
KEGG_INTESTINAL_IMMUNE_NETWORK_FOR_IGA_PRODUCTION	2.376351	1.49E-09
KEGG_LEISHMANIA_INFECTION	2.422107	1.49E-09
KEGG_LYSOSOME	2.306005	1.49E-09
KEGG_NATURAL_KILLER_CELL_MEDIATED_CYTOTOXICITY	2.22178	1.49E-09
KEGG_SYSTEMIC_LUPUS_ERYTHEMATOSUS	2.371002	1.49E-09
KEGG_ANTIGEN_PROCESSING_AND_PRESENTATION	2.254495	1.49E-09
KEGG_AUTOIMMUNE_THYROID_DISEASE	2.310739	1.49E-09
KEGG_TOLL_LIKE_RECEPTOR_SIGNALING_PATHWAY	2.208543	2.49E-09
KEGG_ASTHMA	2.261288	2.82E-09
KEGG_HEMATOPOIETIC_CELL_LINEAGE	2.247546	2.82E-09
KEGG_TYPE_I_DIABETES_MELLITUS	2.292195	3.39E-08
KEGG_B_CELL_RECEPTOR_SIGNALING_PATHWAY	2.055843	4.77E-06
KEGG_NOD_LIKE_RECEPTOR_SIGNALING_PATHWAY	2.061982	8.53E-06
KEGG_RIBOSOME	2.005879	9.43E-06
KEGG_PRIMARY_IMMUNODEFICIENCY	2.116055	1.87E-05
KEGG_VIRAL_MYOCARDITIS	2.011615	2.29E-05
KEGG_LEUKOCYTE_TRANSENDOTHELIAL_MIGRATION	1.930485	4.33E-05
KEGG_FC_GAMMA_R_MEDIATED_PHAGOCYTOSIS	1.927114	6.87E-05
KEGG_PROPANOATE_METABOLISM	−2.08946	9.59E-05
KEGG_BETA_ALANINE_METABOLISM	−2.02228	0.000399
KEGG_DILATED_CARDIOMYOPATHY	−1.82561	0.000409
KEGG_JAK_STAT_SIGNALING_PATHWAY	1.734436	0.000429
KEGG_BUTANOATE_METABOLISM	−1.99342	0.000452
KEGG_SPLICEOSOME	−1.74424	0.00051
KEGG_CYTOSOLIC_DNA_SENSING_PATHWAY	1.863122	0.000978
KEGG_FC_EPSILON_RI_SIGNALING_PATHWAY	1.792667	0.001283
KEGG_ASCORBATE_AND_ALDARATE_METABOLISM	−1.94728	0.001283
KEGG_ARRHYTHMOGENIC_RIGHT_VENTRICULAR_CARDIOMYOPATHY_ARVC	−1.81689	0.001385
KEGG_EPITHELIAL_CELL_SIGNALING_IN_HELICOBACTER_PYLORI_INFECTION	1.802674	0.001432
KEGG_HYPERTROPHIC_CARDIOMYOPATHY_HCM	−1.79619	0.002087
KEGG_PRION_DISEASES	1.805778	0.008099
KEGG_LYSINE_DEGRADATION	−1.79712	0.009665
KEGG_ARGININE_AND_PROLINE_METABOLISM	−1.7153	0.010319
KEGG_FATTY_ACID_METABOLISM	−1.7817	0.010363
KEGG_VALINE_LEUCINE_AND_ISOLEUCINE_DEGRADATION	−1.77887	0.01088
KEGG_T_CELL_RECEPTOR_SIGNALING_PATHWAY	1.626517	0.012534
KEGG_PPAR_SIGNALING_PATHWAY	1.595597	0.015743
KEGG_ARACHIDONIC_ACID_METABOLISM	1.671529	0.015755
KEGG_NEUROACTIVE_LIGAND_RECEPTOR_INTERACTION	1.426851	0.01588
KEGG_VASCULAR_SMOOTH_MUSCLE_CONTRACTION	−1.50513	0.019391
KEGG_BLADDER_CANCER	1.690981	0.024753
KEGG_CARDIAC_MUSCLE_CONTRACTION	−1.51631	0.03298
KEGG_APOPTOSIS	1.520099	0.046472
KEGG_VEGF_SIGNALING_PATHWAY	1.486611	0.047172
KEGG_SULFUR_METABOLISM	1.641416	0.049223
GeneCards_cGAS_STING	1.469654	0.049223

### 3.3. Excharacteristics of DEGs associated with mitochondrial damage and ER stress in early- and late-stage patients

Mitochondrial damage and the ER stress status play important roles in the development of atherosclerosis, so AS patients at different periods might present different transcriptome levels of genes related to mitochondrial damage and ER stress.

**Fig 5:**
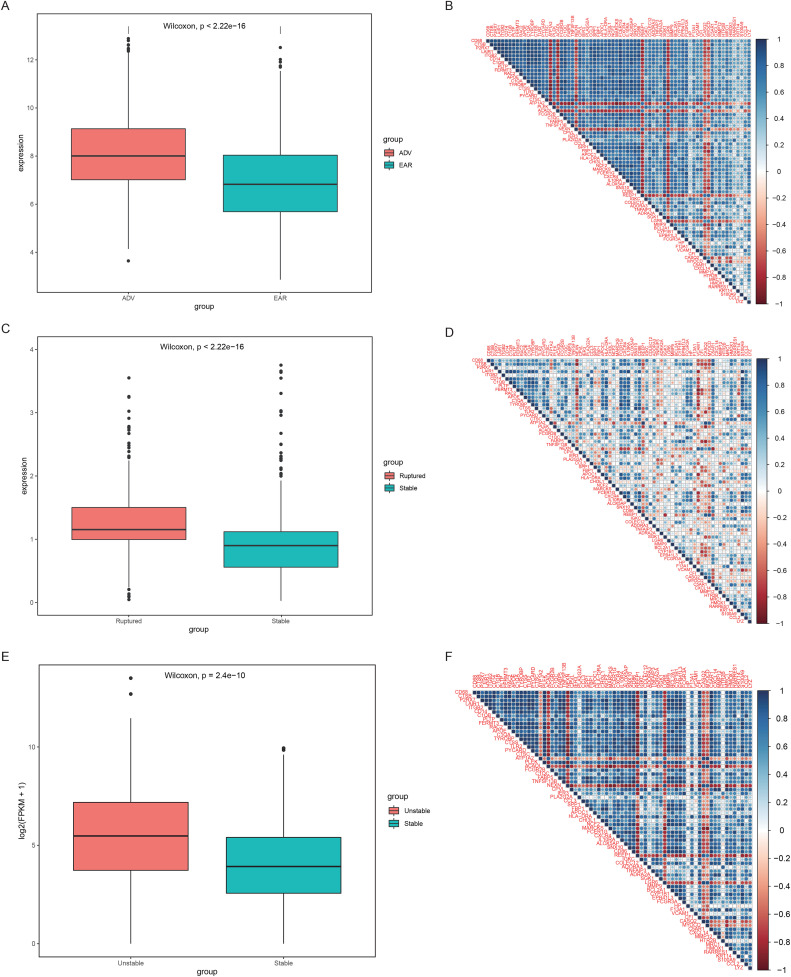
Expression characteristics of genes related to ER stress and mitochondrial damage. **A.** GSE28829 gene expression box plot. The x-axis represents patient groups, the y-axis represents gene expression values, patient groups are distinguished by different colors, the box plot midline represents the median, the upper box represents upper quartiles, the lower box represents lower quartiles, and the statistical test used the Wilcoxon rank sum test. **B.** Heatmap of the GSE28829 gene expression correlation. The color block represents the correlation value; a value less than 0 is blue, that is, a negative correlation, a value greater than 0 is red, or a positive correlation, and the color block area represents the absolute value of the correlation; the larger the absolute value is, the greater the area. **C.** GSE41571 gene expression box plot. **D.** Heatmap of the GSE41571 gene expression correlation. **E.** Box plot of GSE120521 gene expression. **D.** Heatmap of the GSE120521 gene expression correlation.

Therefore, to further analyze the associations among mitochondrial damage, ER stress, and AS status, we grouped patients into early-stage and advanced-stage groups and analyzed genes related to ER stress and mitochondrial damage. Furthermore, we included 2 new datasets to validate our results (GSE41571 and GSE120521, Table 1). Compared with early-stage patients (with stable plaques), advanced-stage patients presented higher expression values of ER stress- and mitochondrial damage-related genes, indicating that ER stress and mitochondrial damage activity were greater in patients with advanced, ruptured, and unstable plaques (Fig 5A, C, E). The correlation heatmap revealed that most of the ER stress and mitochondrial damage genes had strong positive correlations, whereas only a few genes had negative or insignificant correlations (e.g., *CPVL and NEXN*) (Fig 5B). Data from GSE41571 and GSE120521 showed the same correlation features (Fig 5D, F), but the correlation strength was significantly weaker than that of the original dataset, which may be due to the limited number of samples.

### 3.4. Diagnostic model based on ER stress and mitochondrial damage genes

To translate the findings into practical clinical applications, we constructed a diagnostic model to diagnose patients’ status of AS via the expression values of ER stress and mitochondrial damage genes.

**Fig 6:**
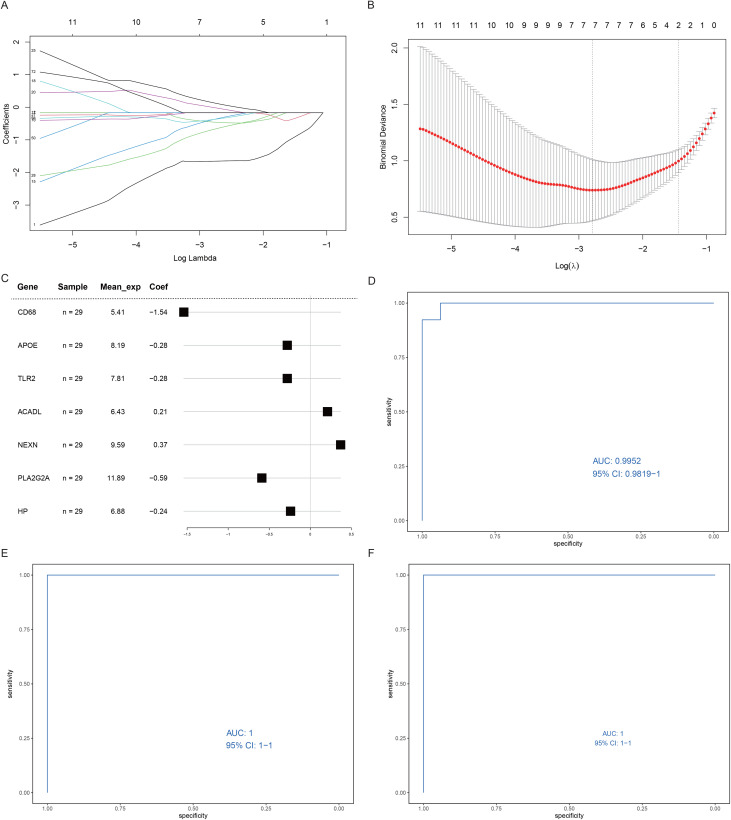
Diagnostic model. A-B. LASSO regression curve showing the convergent screening process of LASSO regression for 72 genes. In Figure A, the x-axis represents the log lambda value, the y-axis represents the regression coefficient, and the lines with different colors represent different features. In Figure B, the abscissa is the tuning parameter (ƛ), the ordinate is the binomial deviation, and the vertical black line indicates the best log value. **C.** Diagnostic model forest plot. The first column shows the seven genes that make up the model, the second column shows the number of samples, the third column shows the average expression values of these genes, and the fourth column and the corresponding figures show the influence coefficients of these genes in the model. **D.** ROC curve of the original dataset. The x-axis represents specificity, and the y-axis represents sensitivity. E-F. ROC curves for the validation datasets GSE41571 and GSE120521.

First, we screened 7 genes from 72 DEGs via least absolute shrinkage and selection operator (LASSO) regression (Fig 6A-B). A diagnostic model containing the 7 genes was subsequently built, and the influence coefficient was defined by its LASSO regression coefficient (Fig 6C). To verify the evaluation efficacy of the diagnostic model, the predicted ROC curves were drawn on the basis of the original datasets GSE 28829 and the external datasets GSE41571 and GSE120521, revealing that the predictive model in both datasets had areas under the curve of 0.9952, 1 and 1 (Fig 6D-F). Overfitting occurred due to the small number of samples in the validation set.

### 3.5. The PPI network of ER stress- and mitochondrial damage-related genes

**Fig 7:**
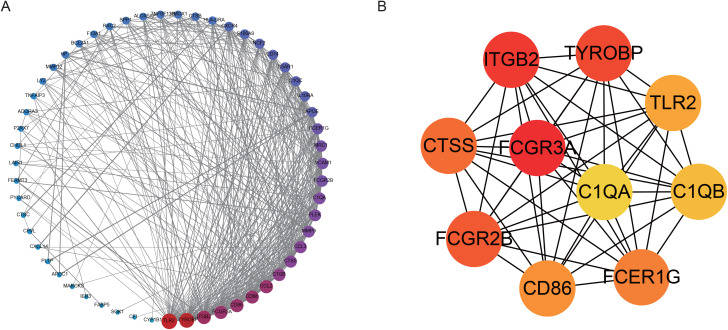
PPI network of ER stress- and mitochondrial damage-related genes. **A.** Network map of 72 ER stress and mitochondrial damage genes. The size of the point is proportional to the degree of the point, the color gradually increases from blue to red, and all nodes are arranged according to the size of the degree. **B.** The 10 hub gene subnetworks. The color of the dot represents the moderate size of the node in the original network A, gradually increasing from yellow to red.

Previous studies have revealed a strong correlation between ER stress and mitochondrial damage, and this correlation might be dominated by a few genes. Therefore, we constructed a PPI network and performed secondary processing to explore these key genes (hub genes). The network contains 72 DEGs related to ER stress and mitochondrial damage, consisting of 381 edges or 381 interaction pairs, and all nodes are colored and arranged in circles to discover the hub genes (Fig 7A) more intuitively in the network. The plugin cytoHubba in Cytoscape was used for hub gene mining with the MCC algorithm standard, and the 10 nodes with an extraction degree of the top 10 were considered hub genes (Fig 7B). These genes were *FCGR3A, ITGB2, TYROBP, FCGR2B, CTSS, FCER1G, CD86, TLR2, C1QB*, and *C1QA*.

### 3.6. Hub gene correlation network prediction

**Fig 8:**
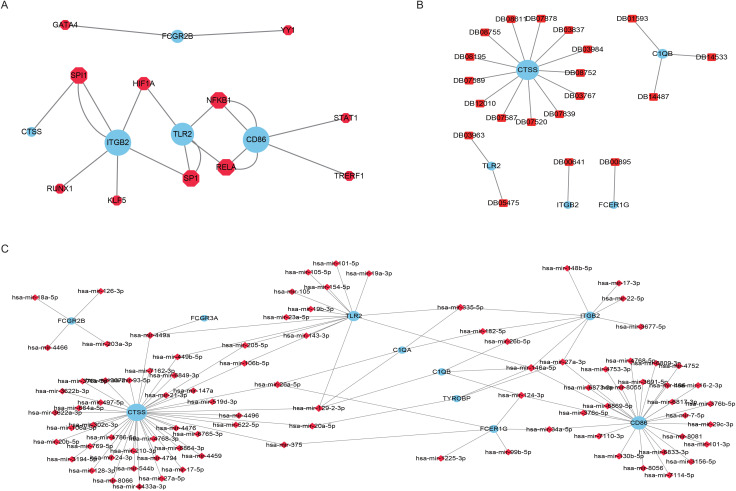
Hub gene network prediction. **A.** Hub gene–A transcription factor network. The blue circular nodes are hub genes, and the red polygonal nodes are transcription factors. **B.** Hub gene–drug small-molecule network. The blue round nodes are hub genes, and the red square nodes are small drug molecules. **C.** Hub gene‒miRNA network. The blue circular nodes are hub genes, and the red diamond nodes are miRNAs.

Hub genes often play important roles in biological processes, so their interaction with other biomolecules, such as miRNAs and TFs, which also have great potential for use as small-molecule drug targets, is also more active. Therefore, we predicted miRNAs and transcription factors (TFs) associated with 10 hub genes via the miRNet database, identified small molecule drugs that interact with hub genes via the DrugBank database, and generated related subnetworks via Cytoscape software. The Hub-TF network consists of 20 interaction pairs and 11 TFs (Fig 8A), the Hub-drug small molecule network contains 20 interaction pairs and 20 drug small molecules (Fig 8B), and the Hub-mi RNA network contains 115 interaction pairs and 90 miRNAs (Fig 8C).

### 3.7. Correlations between Hub genes and immune infiltration

To further explore the relationships between the hub genes and the level of infiltration of immune cells, we calculated immune cell infiltration scores for all the samples on the basis of the 28 immune cell background gene sets via the ssGSEA method. On the basis of the expression levels of the 10 hub genes in all patients, the patients were divided into two groups of high and low-expression groups, with 16 and 13 cases, respectively.

**Fig 9:**
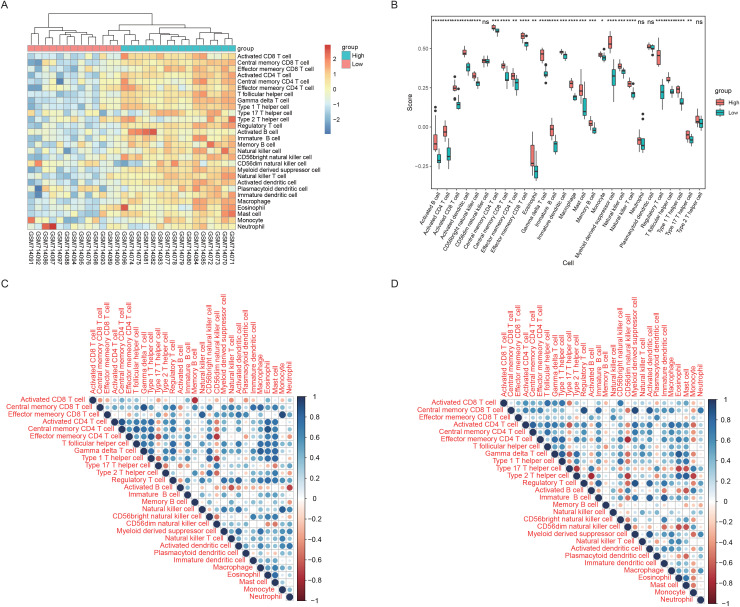
ssGSEA assessment of immune infiltration. A. Heatmap of the immune score. The top color bars represent two groups: red represents low expression, green represents high expression, blue represents low immune infiltration, and red represents high immune infiltration. B. Immune-scoring box plot. The x-axis shows 28 immune cells, the y-axis shows the level of immune infiltration, each color represents a patient grouping, and the statistical test is the Wilcoxon rank-sum test. The upper symbols represent the significance level of the difference, * less than 0.05, * * 0.01, * * * 0.001, * * * 0.0001 * *, ns and unsigned represent nonsignificant differences. C. Correlation heatmap of 28 immune cells in patients in the high-expression group. D. Correlation heatmap of the 28 immune cells tested in patients in the low-expression group.

The results revealed that up to 24 of the 28 immune cells were significantly different between the two groups, indicating a strong correlation between the hub genes and immune processes. In addition, almost all immune cells in the high-expression group had a high infiltration fraction (Fig 9A-B), indicating that the active state of ER stress and mitochondrial damage were correlated with the activation of immune processes, which was completely consistent with the results of previous gene enrichment analyses. Moreover, these findings also illustrated the accuracy and effectiveness of the hub gene screening, as the expression levels of these genes could significantly distinguish between immune statuses.

Correlations between pairwise immune cells were calculated. The results revealed that the correlation between immune cells was relatively stable in both groups. For example, three CD4 T cells were positively correlated in both groups, whereas effector memory CD8 T cells and activated CD8 T cells were negatively correlated in the high-expression group but positively correlated in the low-expression group, indicating distinct characteristics of ER stress and mitochondrial damage (Fig 9C-D).

**Fig 10:**
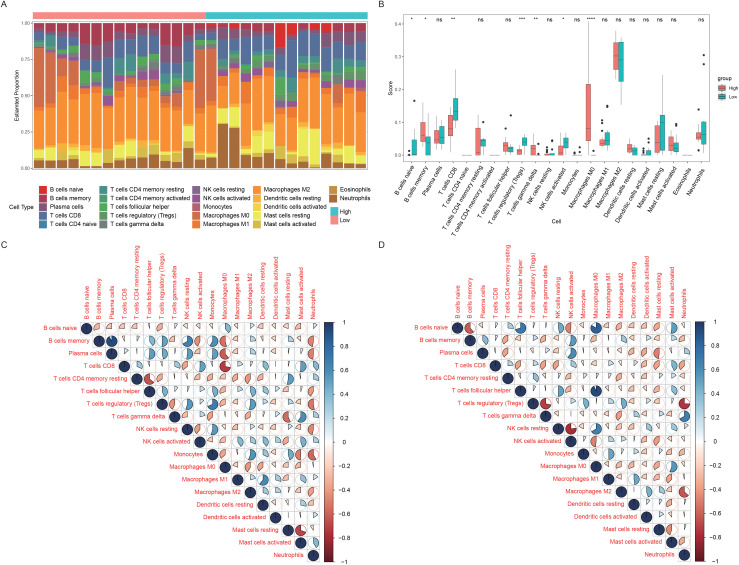
CIBERSORT assessment of immune infiltration. **A.** Bargram of immune scores. The x-axis represents 29 patients, the y-axis represents the immune cell proportion, each color represents one immune cell, the column length represents the proportion of immune cells in the whole population, green represents patients in the high-expression group, and red represents patients in the low-expression group. **B.** Immune-scoring box plot. The x-axis shows 22 immune cells, the y-axis shows the immune infiltration level, each color represents a patient grouping, and the statistical test is the Wilcoxon rank-sum test. The upper symbols represent the significance level of the difference, * less than 0.05, * * 0.01, * * * 0.001, * * * 0.0001 * *, ns and unsigned represent nonsignificant differences. **C.** Correlation heatmap of the 22 immune cells tested in the high-expression group of patients. **D.** Related heatmap of 22 immune cells in the low-expression group.

To further validate our conclusions, another commonly used immune infiltration assessment method—cibersort—was calculated based on 22 immune cell background gene sets. We found that the infiltration levels of 7 of the 22 immune cells, especially M0 macrophages, were significantly different between the two groups, with almost 0 infiltrating immune cells in the low-expression group and remarkably high infiltration levels in the high-expression group. Consistent with the results of ssGSEA, the infiltration levels of most immune cells were greater in the high-expression group (Fig 10A-B). Similarly, the correlation of each immune cell type in both groups was relatively stable. However, some cells, such as naive B cells and activated mast cells, were negatively correlated in the high-expression group but positively correlated in the low-expression group (Fig 10C-D).

**Fig 11:**
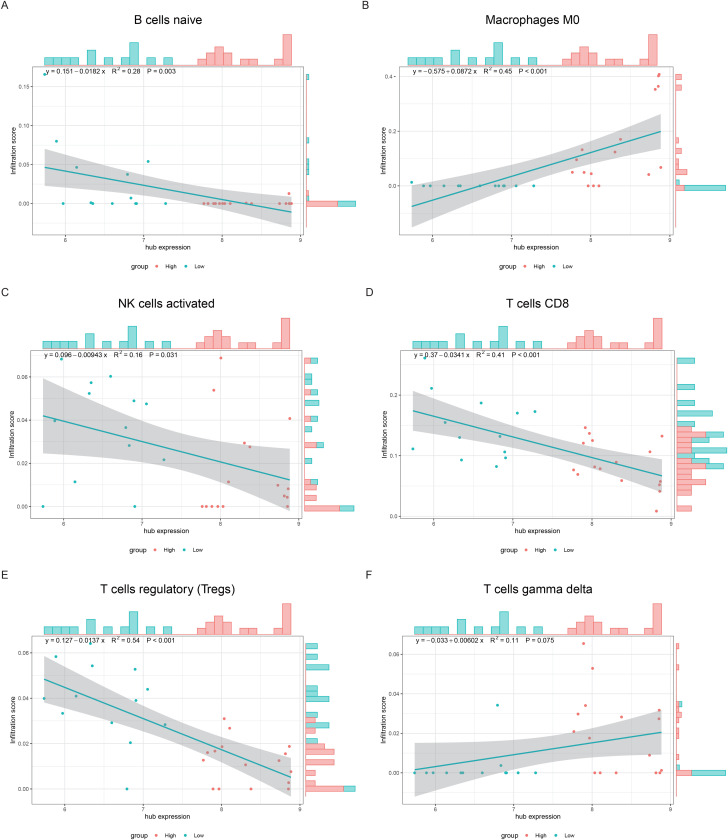
Scatter plot of the correlation of the hub gene expression values versus immune cells vs. A-F is B-cell naive, M0 macrophages, activated NK cells, CD8+ T cells, regulatory T cells (Tregs), and gamma delta T cells. The x-axis represents the mean of all hub genes expressed. The y-axis represents the level of immune cell infiltration, and each point represents a single patient sample. Each color represents a single patient group. The straight line in the figure shows the correlation fitting curve, and the shaded sections represent the confidence intervals. The outside of the figure is the corresponding histogram.

Finally, to directly determine the relationships between the hub genes and immune cell infiltration, the expression levels of 6 cell types (naive B cells, M0 macrophages, activated NK cells, CD8 T cells, regulatory T cells (Tregs), and gamma delta T cells) from the CIBERSORT results and hub gene expression levels were used to construct scatter plots and correlation curves. The results revealed that all cells (except gamma delta T cells) presented a direct and significant correlation with the hub gene expression value. The average expression values of the hub genes were positively correlated with M0 macrophages but negatively correlated with the other genes (Fig 11).

## 4. Discussion

The pathogenesis of atherosclerosis (AS) remains elusive, but studies suggest that it involves multiple biological processes, such as endoplasmic reticulum stress, mitochondrial damage, inflammatory responses, and lipid metabolism abnormalities [1,27,28]. Recent discoveries emphasize the significant roles of endoplasmic reticulum stress and mitochondrial damage in AS progression. The endoplasmic reticulum is integral to cellular lipid and protein synthesis, and stress in the endoplasmic reticulum can lead to lipid metabolism abnormalities, influencing AS-related inflammation. Mitochondria, which are essential for cellular energy metabolism and oxidative stress, can contribute to inflammation and immune responses in AS following damage [3].

Different biomarkers models for risk stratification have been found to carry prognostic information in CVDs. Tradtional lipid and metabolite biomarkers have been dicussed a lot in the past years. PLATO study also found higher levels of GDF-15 and NT-proBNP were associated with raised risks of bleeding, spontaneous MI, and stroke as well as CV death. However, a single molecule is not completely suitable for diagnosis, especially at an early stage. In a recent paper by Siegbahn et al, a CVD risk model consisting of 21 biomarkers was developed aiming at improvement of risk stratification, decision support, and treatment precision in patients with different CVDs. This provides a novel way to explore modalities for treatment and prevention using multiplex protein analyses. However, early detection, primary prevention of atherosclerosis progress, have been long-standing scientific research goals. To our knowledge, this is the first time that involves endoplasmic reticulum stress, mitochondrial damage into a diagnosis tool for better treatment and monitoring of CVD.

By mining published public data, such as the GSE28829 dataset from the GEO database, we investigated potential mechanisms underlying AS, contributing to clinical diagnosis and treatment. To explore the impact of endoplasmic reticulum stress and mitochondrial damage on AS, we analyzed 72 DEGs related to these conditions. GO enrichment analysis revealed pathways such as neutrophil activation and degranulation, indicating a significant correlation with neutrophil-mediated immune processes. KEGG enrichment analysis further highlighted cell immunity pathways. For clinical translation, we employed LASSO regression to refine these genes, constructing a diagnostic model for AS validated by receiver operating characteristic (ROC) curve analysis, which showed excellent predictive efficiency. Additionally, protein–protein interaction (PPI) analysis revealed 10 hub genes (*FCGR3A, ITGB2, TYROBP, FCGR2B, CTSS, FCER1G, CD86, TLR2, C1QB, and C1QA*) involved in AS pathogenesis.

Therefore, the hub genes we identified might play critical roles in the onset and progression of atherosclerosis. The development of atherosclerosis is commonly associated with inflammatory responses, immune cell responses, and macrophage phagocytosis. For example, our findings indicate that the FCGR3A gene encodes FcγRIIIa, a low-affinity receptor for the Fc region of immunoglobulin G (IgG). The literature suggested FCGR3A(CD16A) + monocytes were significantly increased in apoE-/- mice with high fat diet, which might correlate with aortic MMP-9 mRNA expression and serum TNF-α level, indicaitng FCGR3A involved in the progression of atherosclerosis through inflammatory pathways. [29,30]. The ITGB2 gene encodes integrin β2, which is crucial for the adhesion and migration of immune cells. Moreover, as a ROS-related protein, ITGB2 could interact with multiple genes (e.g., HIF-1α) to promote ROS production, NLRP3 inflammasome activation, pyroptosis, and foam cell formation in macrophages, suggesting its potential role in the progression of atherosclerosis [31]. CTSS and TLR2 might be involved in the modulation of vascular repair processes through their involvement in the p38MAPK signaling pathway [32]. TYROBP (DAP12) regulates phosphorylation of multiple signaling mediators, and positively correlates to neutrophil chemotaxis, macrophage activation, chemokine signaling pathway, JAK-STAT signaling pathway. TIMER2.0 and ssGSEA showed that TYROBP expression was significantly associated with the infiltration of neutrophils, macrophages, myeloid dendritic cells and monocytes, thereby potentially influencing the progression of atherosclerosis [33]. Other genes, such as FCER1G, might influence the formation of atherosclerotic plaques through antibody responses, while CD86 might affect atherosclerosis by mediating T cells/DCs activation. [34,35]. Finally, the C1QB and C1QA genes regulate the development of atherosclerosis by activating the complement system [36].

In the subsequent phase of our research, utilizing the DrugBank database, we successfully identified small molecule drugs that interact with these genes. By employing Cytoscape for visualization, we elucidated potential insights for clinical therapy. This approach not only highlights the therapeutic relevance of our findings but also paves the way for future investigations into targeted drug development for the treatment of related conditions. However, like any other models, our research has its limitations. Since the insufficient sample in our datasets, overfitting may be difficult to eliminate. This may lead to increased risk of false positives or false negatives, limiting its usefulness in real-world applications.

Furthermore, studies have revealed a relationship between m6A modification and AS [37]. Our investigation revealed a positive correlation between m6A genes and hub genes (S1-S4 Figs and S1 Text). By analyzing the relationships between hub genes and immune cell infiltration, we found a strong association with immune processes. Additionally, the STING pathway was highly correlated with the hub genes involved in AS development (S5 Fig and S1 Text), indicating its promising role as a potential therapeutic target in AS, which is consistent with our recent studies [38–42]. In summary, this study revealed hub genes related to ER stress and mitochondrial damage in the pathogenesis of atherosclerosis (AS). These hub genes offer new directions for further research into disease mechanisms and the development of pharmacological treatments.

## 5. Summary

Studies have emphasized the significant roles of endoplasmic reticulum stress and mitochondrial damage in AS progression. By mining published public data, we investigated potential mechanisms underlying AS, contributing to clinical diagnosis and treatment. Seventy-two DEGs were related to endoplasmic reticulum stress and mitochondrial damage. PPI analysis identified 10 hub genes (*FCGR3A, ITGB2, TYROBP, FCGR2B, CTSS, FCER1G, CD86, TLR2, C1QB, and C1QA*) involved in AS pathogenesis. For clinical translation, we employed LASSO regression to refine these genes, constructing a diagnostic model for AS validated by receiver operating characteristic (ROC) curve analysis, which showed excellent predictive efficiency. Additionally, GO enrichment analysis revealed pathways such as neutrophil activation and degranulation, indicating a significant correlation with neutrophil-mediated immune processes. KEGG enrichment analysis further highlighted cell immunity pathways. We subsequently successfully identified small molecule drugs that interact with these genes via the DrugBank database. By employing Cytoscape for visualization, we elucidated potential insights for clinical therapy. Our investigation revealed a positive correlation between m6A genes, STING pathways and hub genes, indicating its promising role as a potential therapeutic target in AS. In summary, this study revealed hub genes related to ER stress and mitochondrial damage in the pathogenesis of atherosclerosis (AS). These hub genes offer new directions for further research into disease mechanisms and the development of pharmacological treatments.

## Supporting information

S1 Fig Correlation analysis between the m6A gene and the hub gene. A. Heatmap of Pearson correlation between expression levels of Hub gene and m6A genes. B. Significant interaction network map of Pearson correlation for expression levels of Hub genes and m6A genes. Red dots represent the m 6A gene, and green dots represent the hub gene. Point size represents the connectivity, and line thickness represents the Pearson correlation size.
(PDF)

S2 Fig m6A regulator expression and localization. A. Heatmap of the m6A regulon expression in early and late atherosclerosis. B. Box plot of the expression of the m6A regulon in early and late atherosclerosis. C. Information on the position of the m6A regulators on the chromosome.
(PDF)

S3 Fig Pearson correlation for the expression of m6A regulators. **A-C** are the Pearson correlation coefficients of the significantly different m6A regulators in the GSE 22829 and in the validation sets GSE41571, GSE120521, respectively.
(PDF)

S4 Fig Type-typing analysis based on m6A regulators. A, C, and E are the classification index plots of the sample prediction for GSE 28829, GSE 41571, and GSE120521 based on significantly different m6A regulators, respectively. **B, D, and F** are the clustering results of NMF on the GSE 28829, GSE 41571, and GSE 120521 datasets, respectively.
(PDF)

S5 Fig Correlation between hub gene expression and the expression of genes associated with cGAS-STING, STING-IRF3, and STING-NLRP3. **A.** Heatmap of the correlation between Hub genes and genes associated with cGAS-STING. Red represents a positive correlation, and blue represents a negative correlation. **B.** Circular plot of the correlation of Hub genes and genes associated with STING-IRF3. The wired color between genes in the figure represents the degree of correlation, red represents the positive correlation, green represents the negative correlation, and color depth represents the correlation size. **C.** Lollipop plot of correlation between Hub gene and STING-NLRP3 related genes. The abscissa represents the hub gene, the ordinate represents the correlation with the NLRP3 gene, and the master height represents the correlation size.
(PDF)

S1 Text Relationship between hub genes and m6A regulators, STING pathways. (DOCX)
