# Supplementary figures and images for: Identification and validation of diagnostic markers of atherosclerosis progression via bioinformatics strategies
Source: PLoS One. 2025 Dec 5;20(12):e0336139. doi: 10.1371/journal.pone.0336139 (PMC12680236; doi:10.1371/journal.pone.0336139)

A

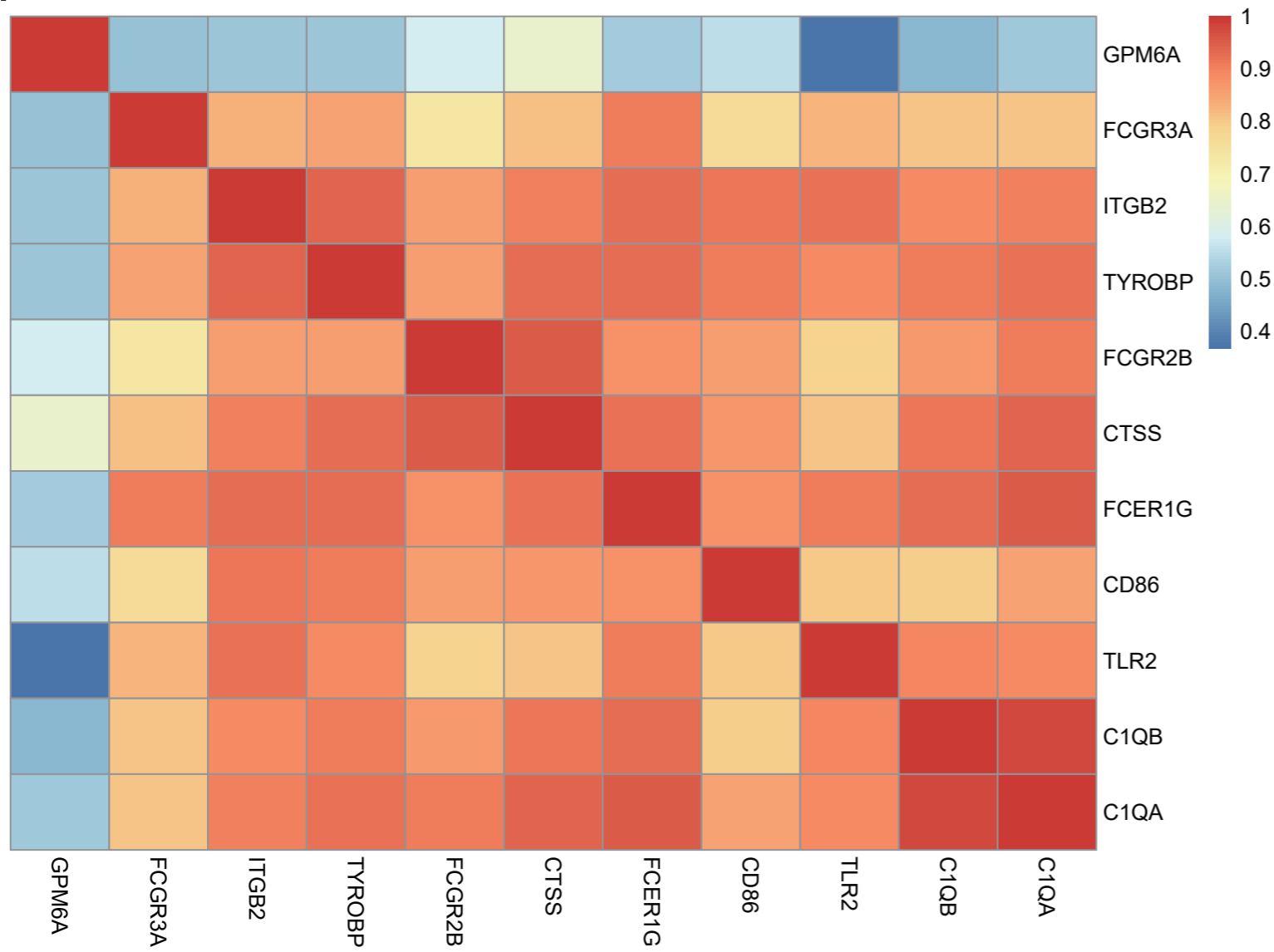

B

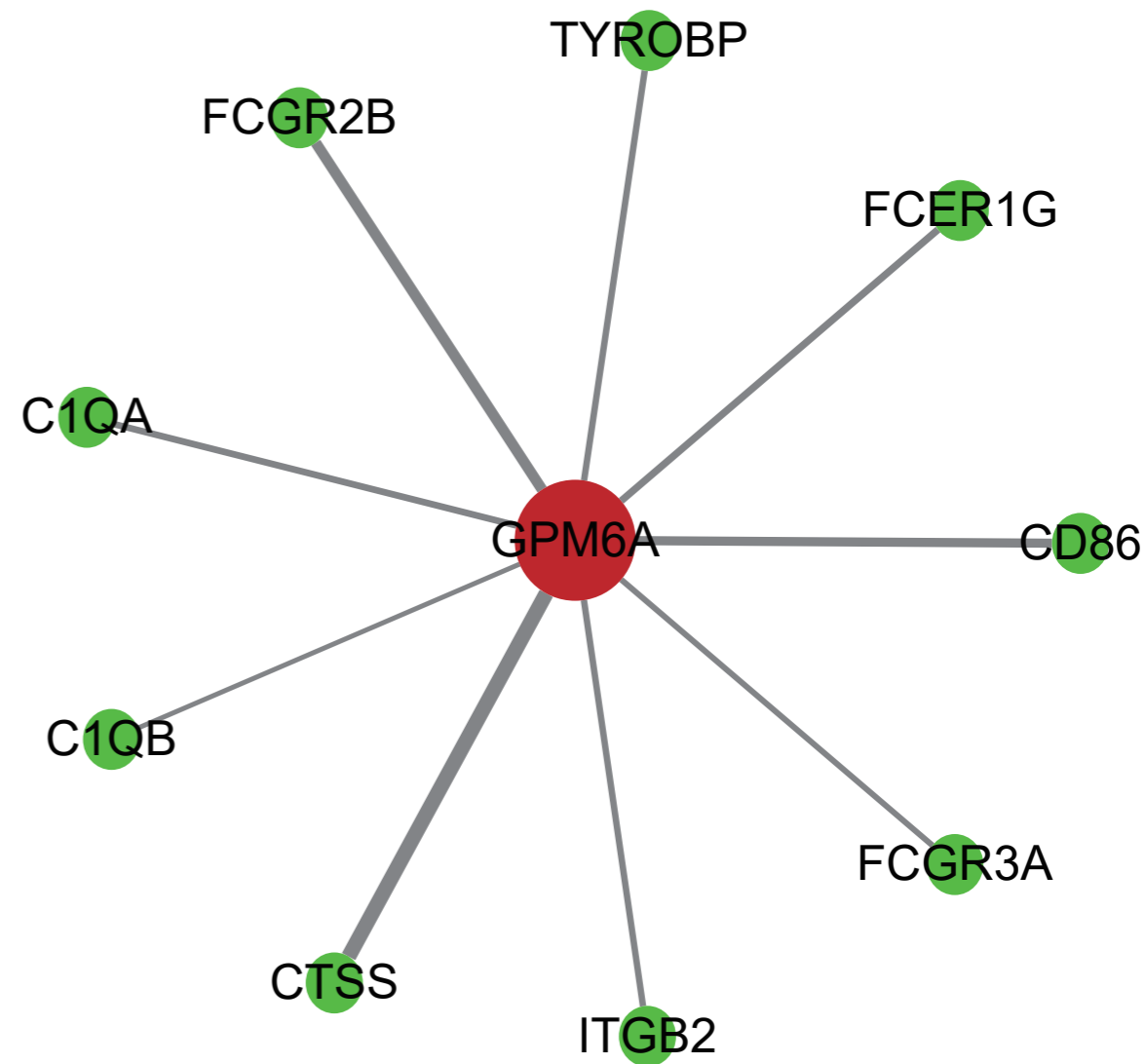

Supplement: S1 Fig — A. Heatmap of Pearson correlation between expression levels of Hub gene and m6A genes. B. Significant interaction network map of Pearson correlation for expression levels of Hub genes and m6A genes. Red dots represent the m 6A gene, and green dots represent the hub gene. Point size represents the connectivity, and line thickness represents the Pearson correlation size. (PDF) [file pone.0336139.s001.pdf]

A

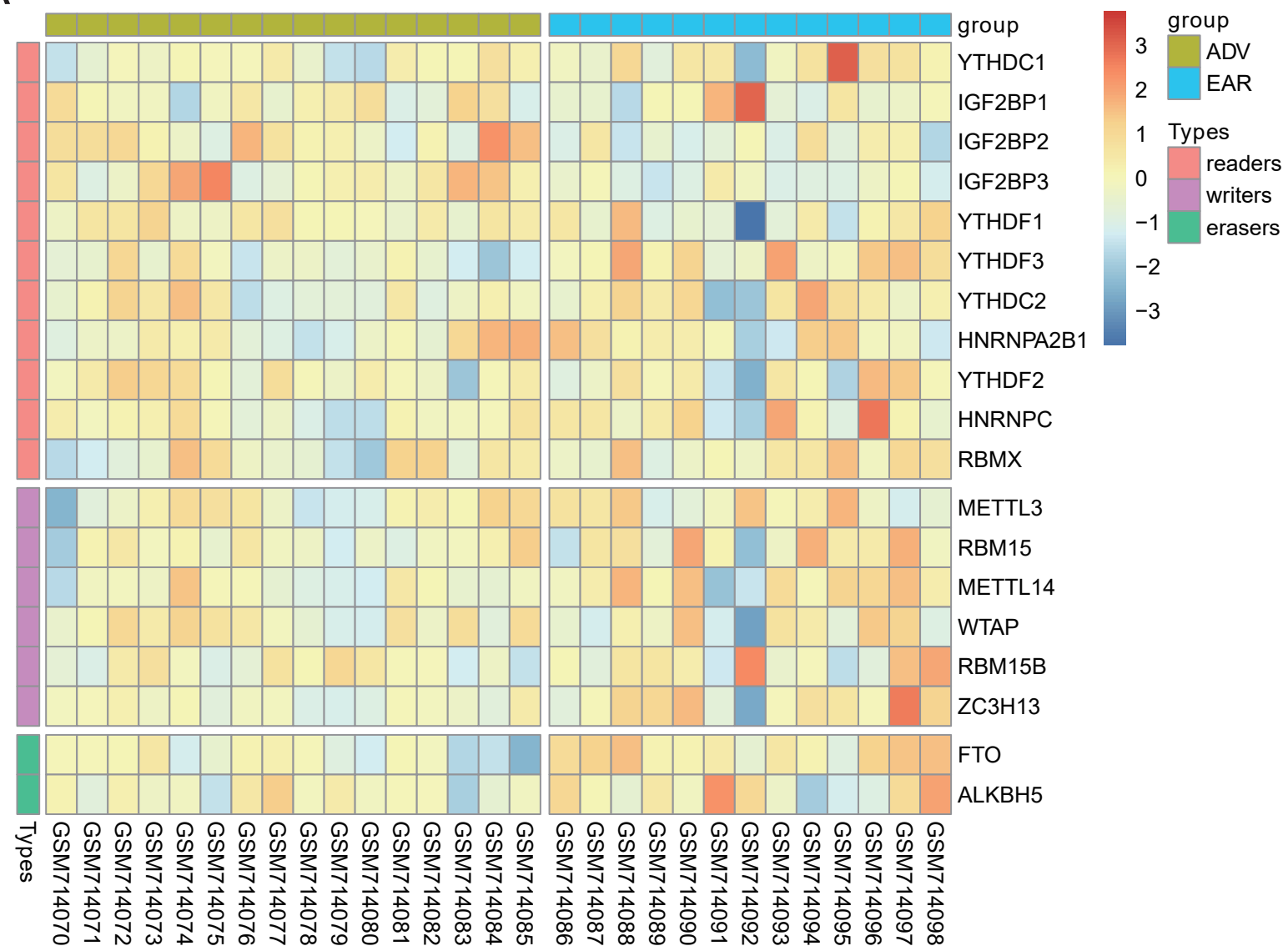

B

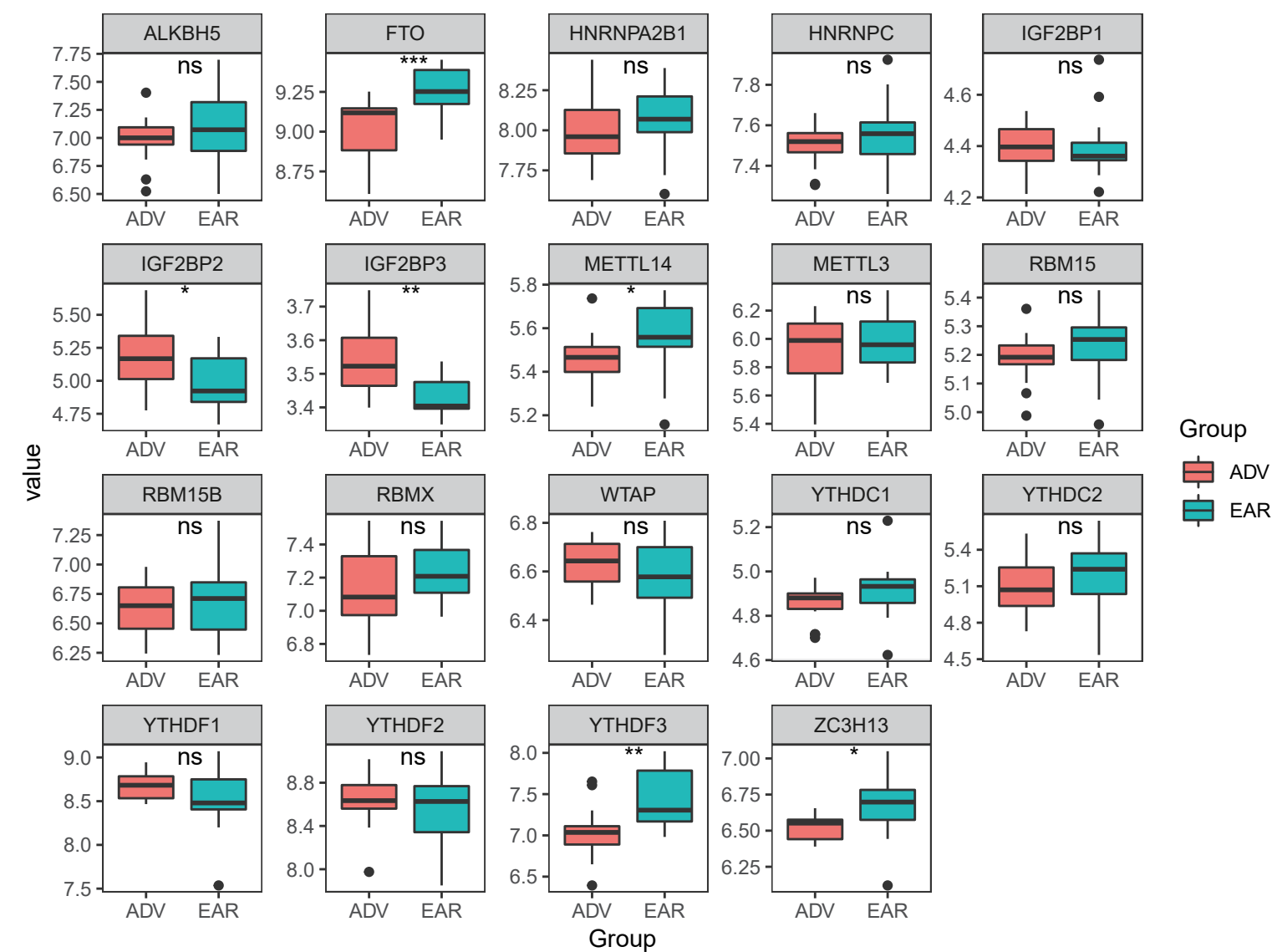

C

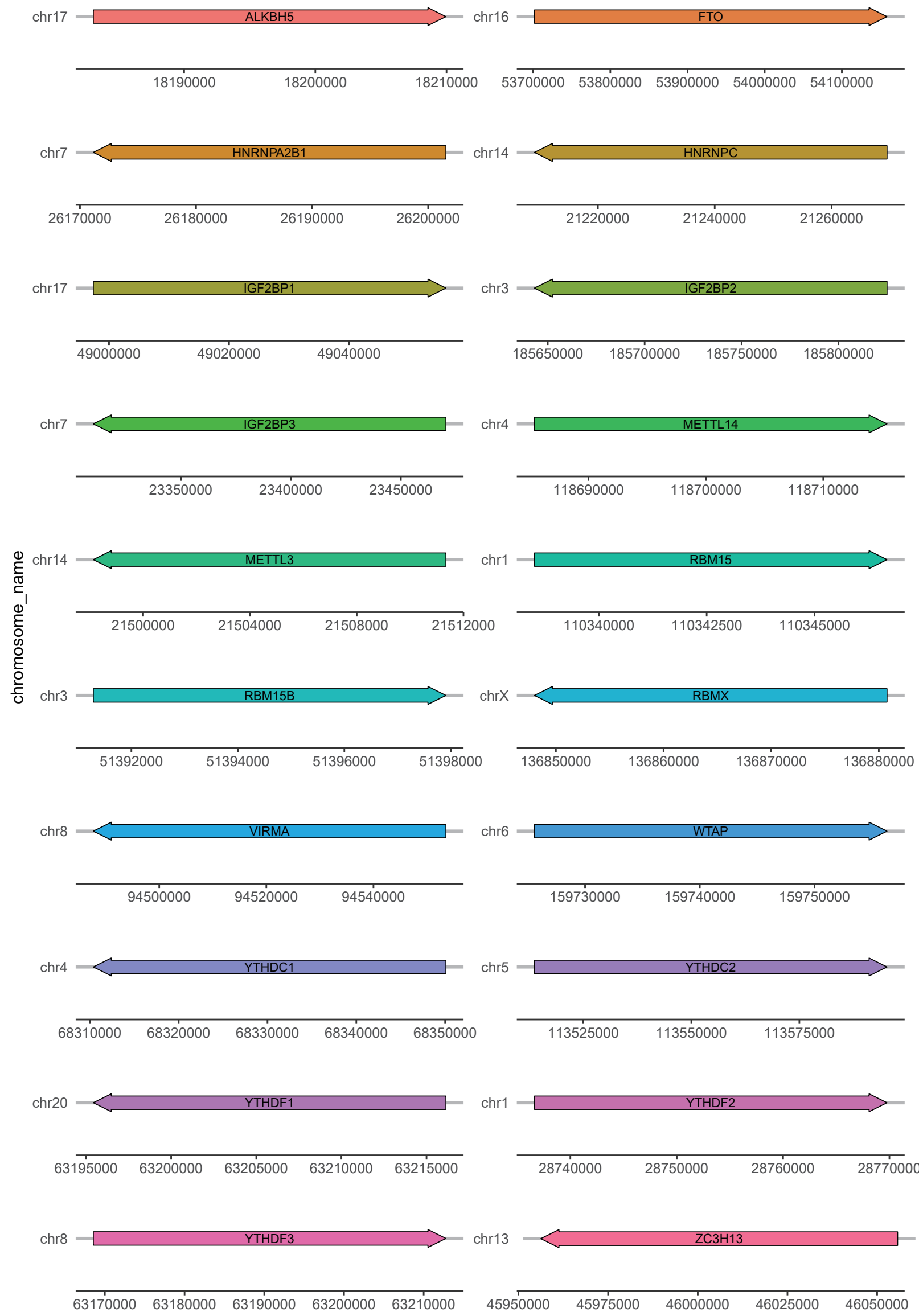

Supplement: S2 Fig — A. Heatmap of the m6A regulon expression in early and late atherosclerosis. B. Box plot of the expression of the m6A regulon in early and late atherosclerosis. C. Information on the position of the m6A regulators on the chromosome. (PDF) [file pone.0336139.s002.pdf]

A

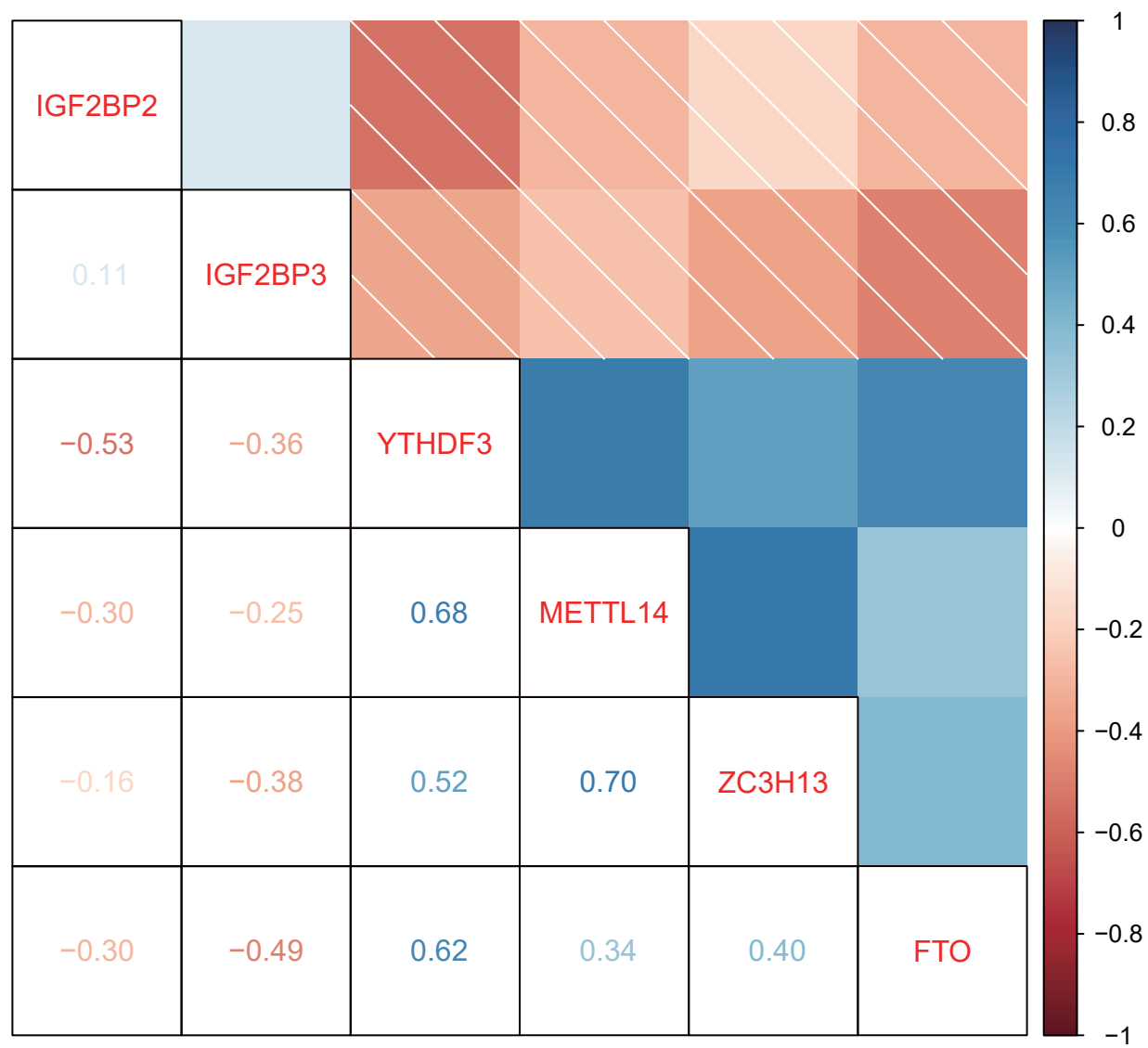

B

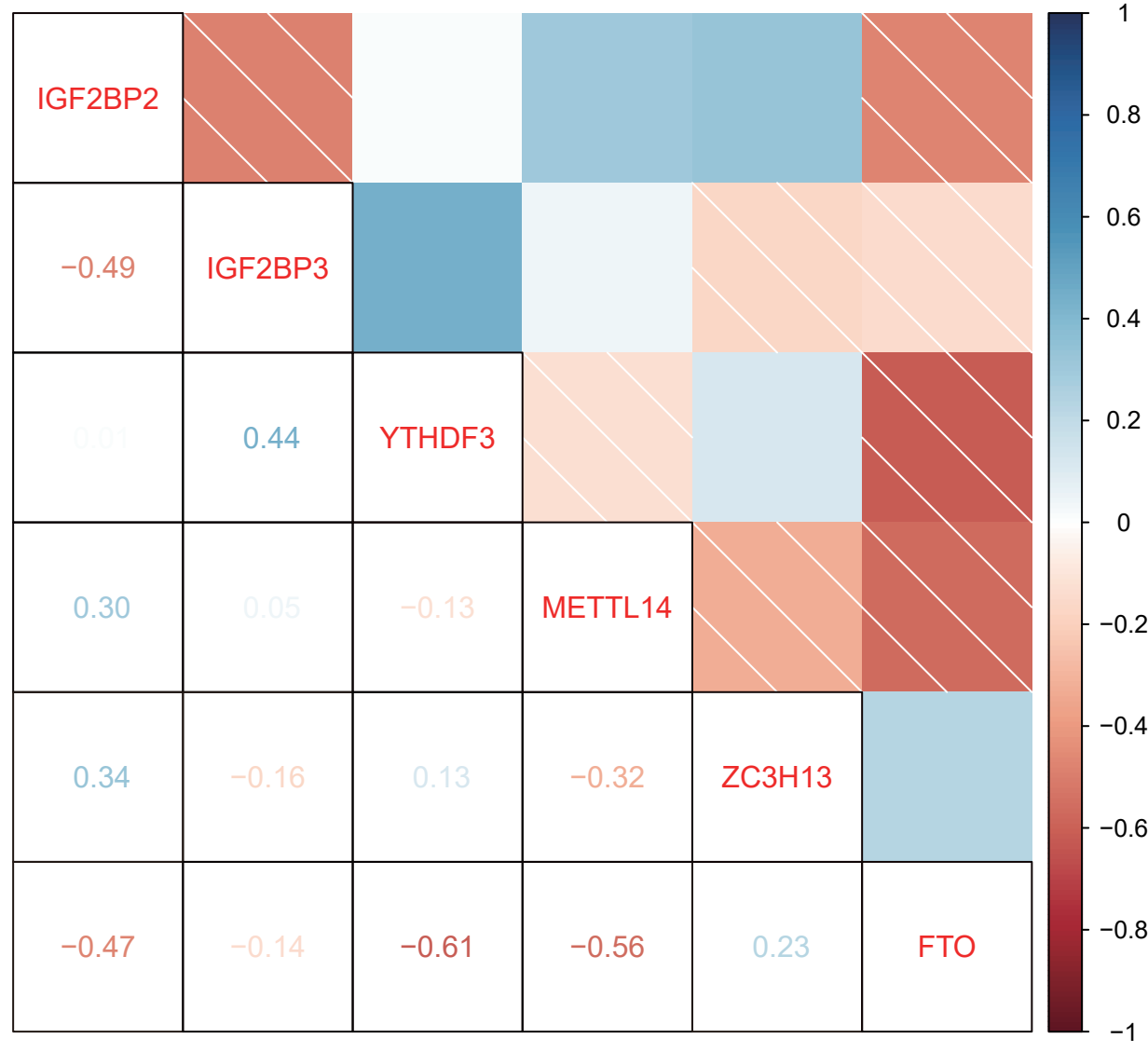

C

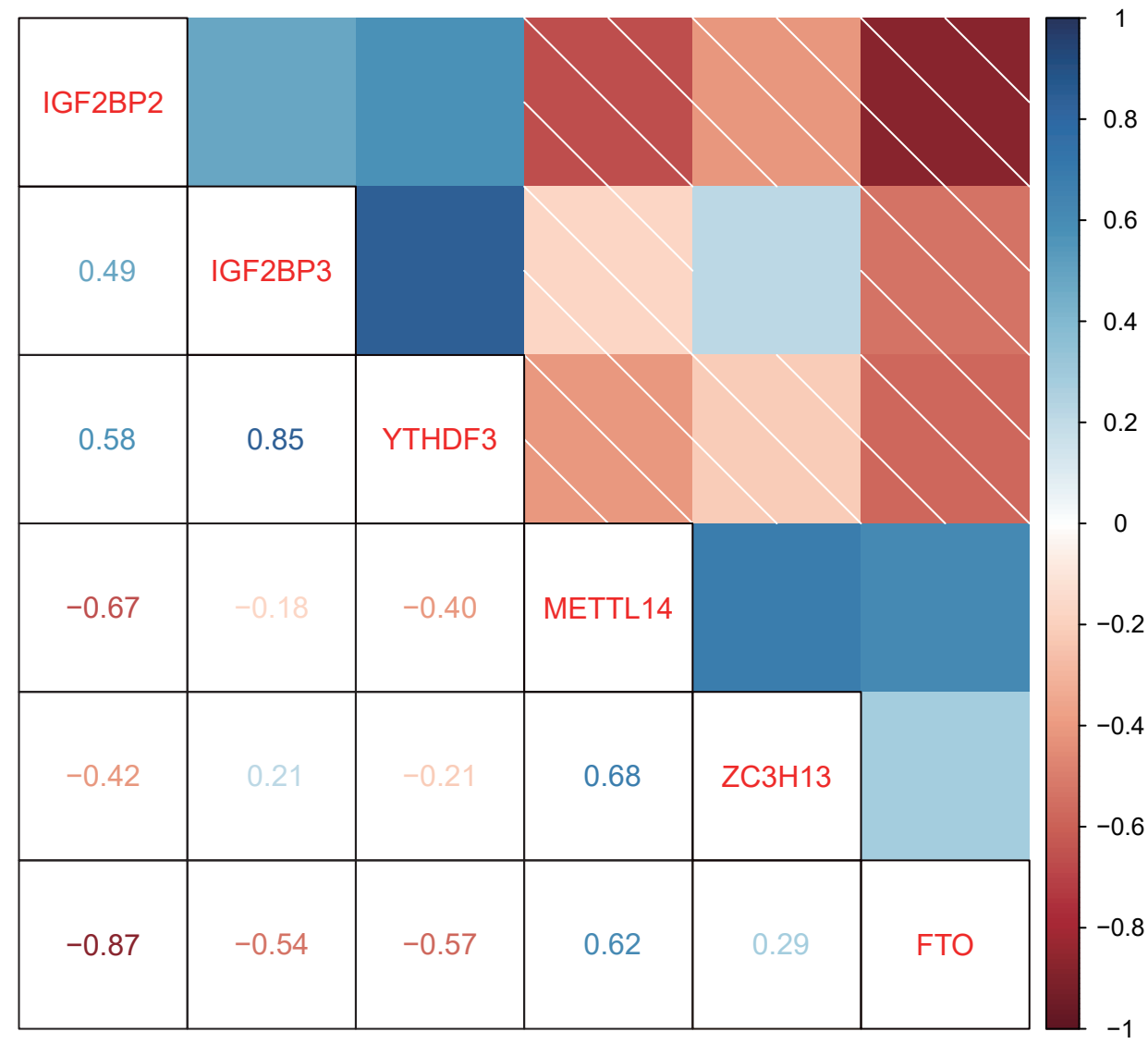

Supplement: S3 Fig — A-C are the Pearson correlation coefficients of the significantly different m6A regulators in the GSE 22829 and in the validation sets GSE41571, GSE120521, respectively. (PDF) [file pone.0336139.s003.pdf]

A

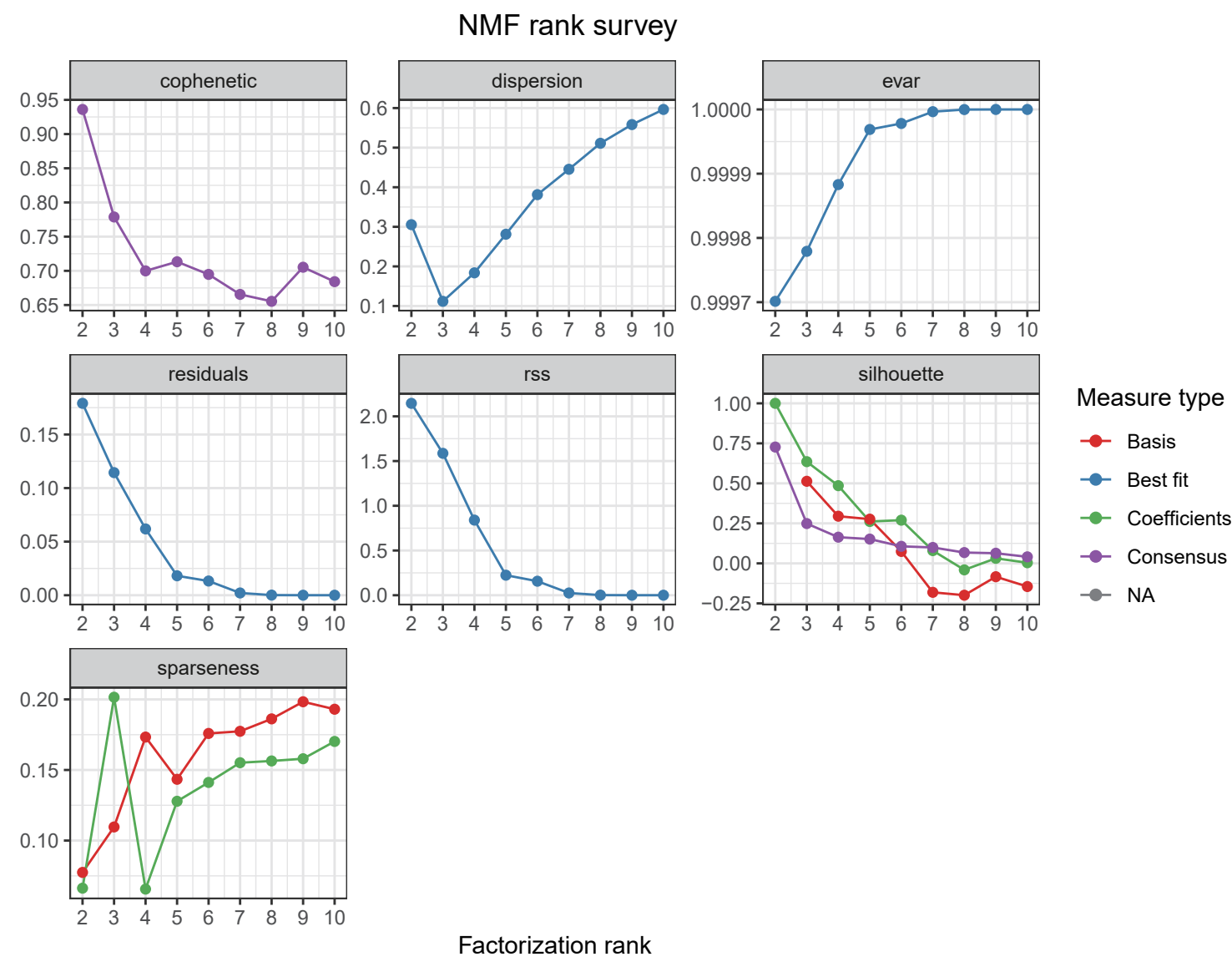

B

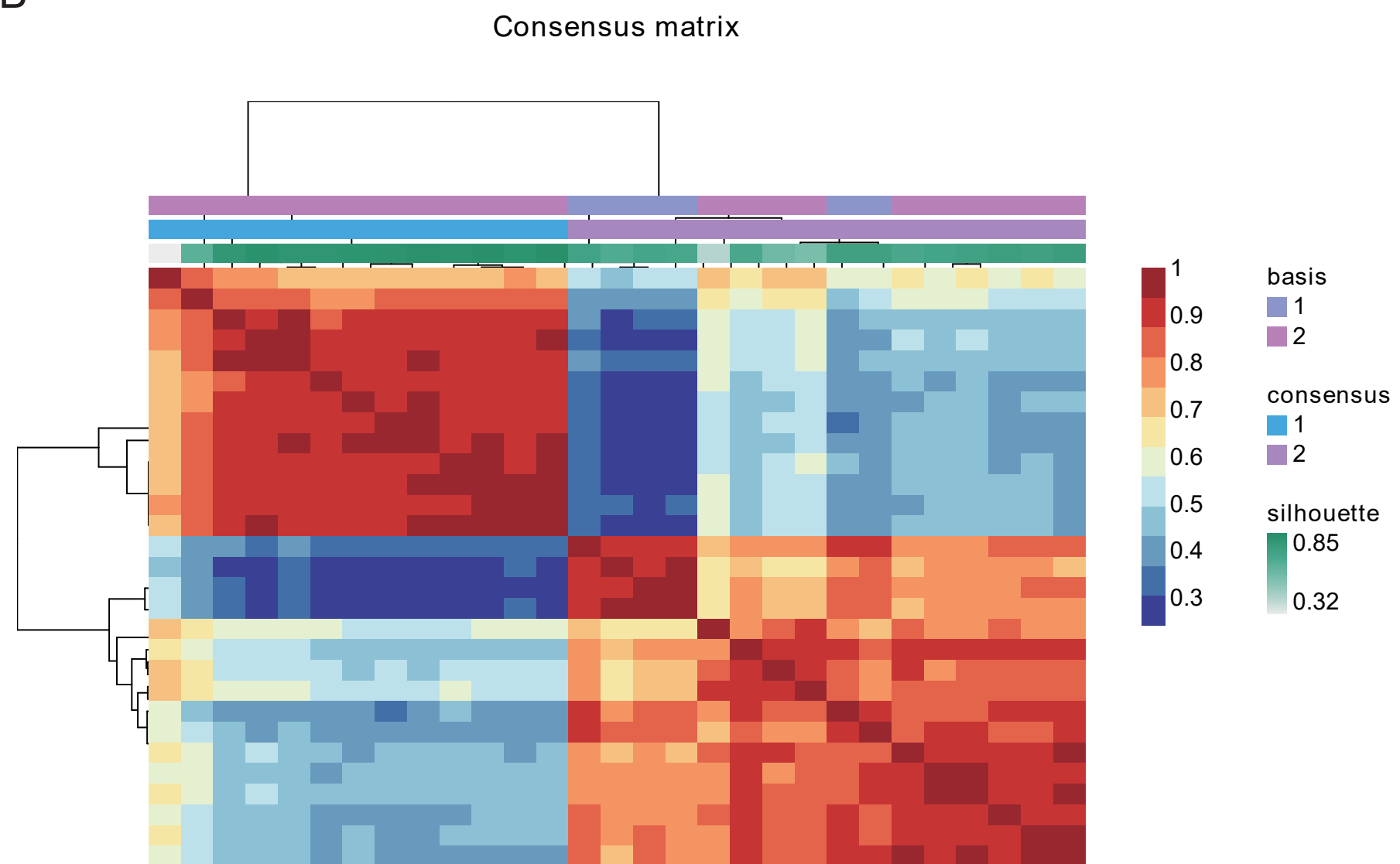

C

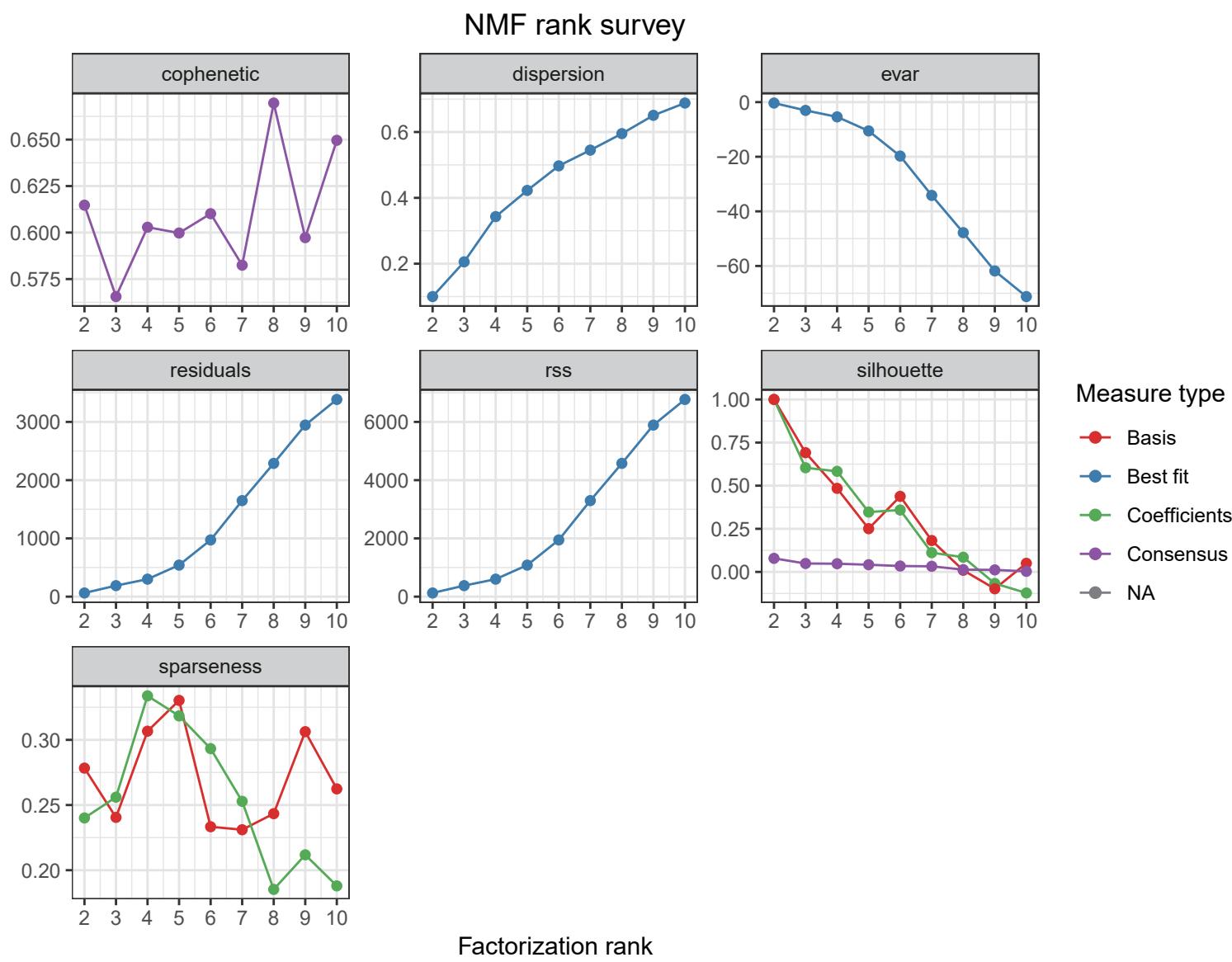

D

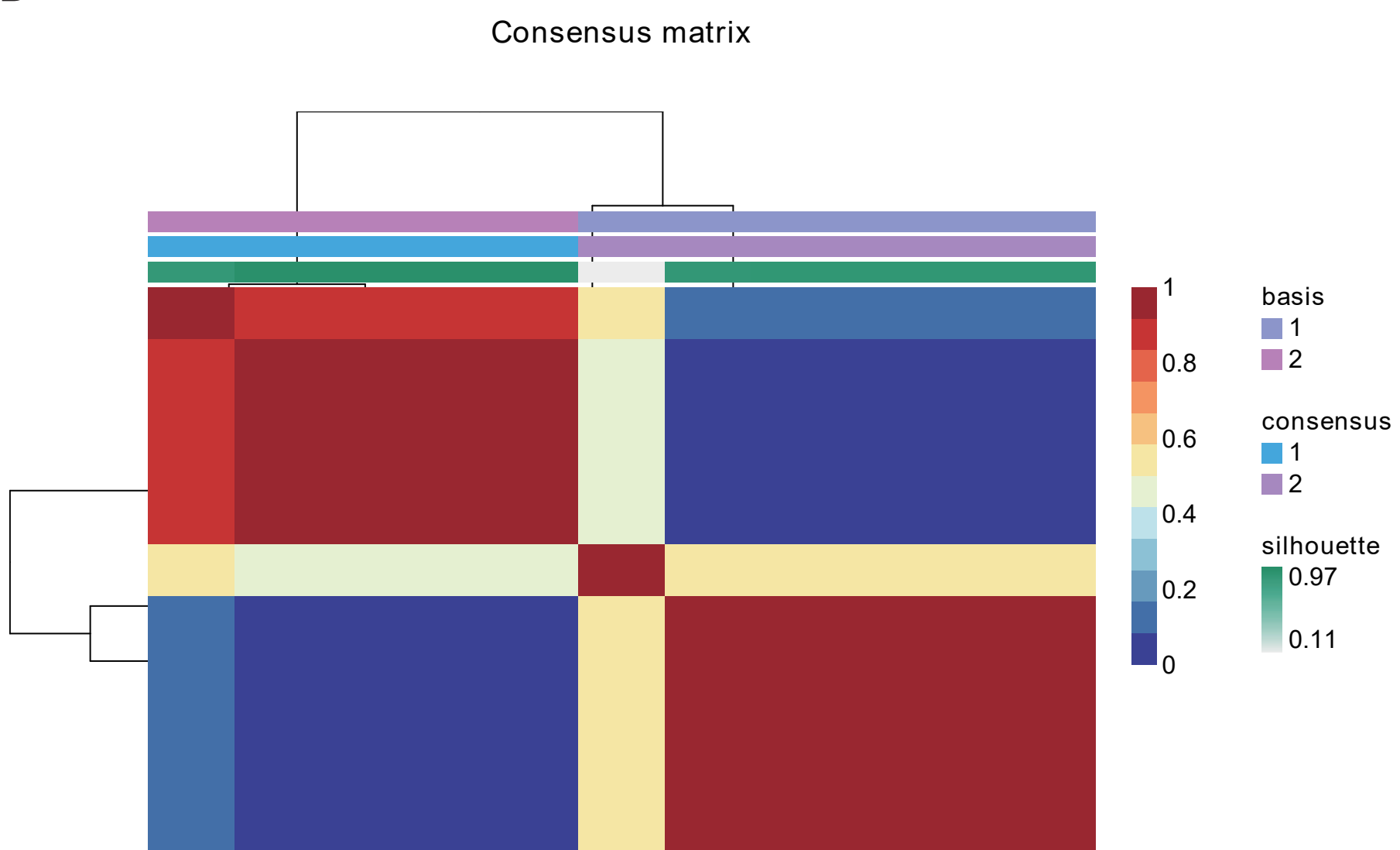

E

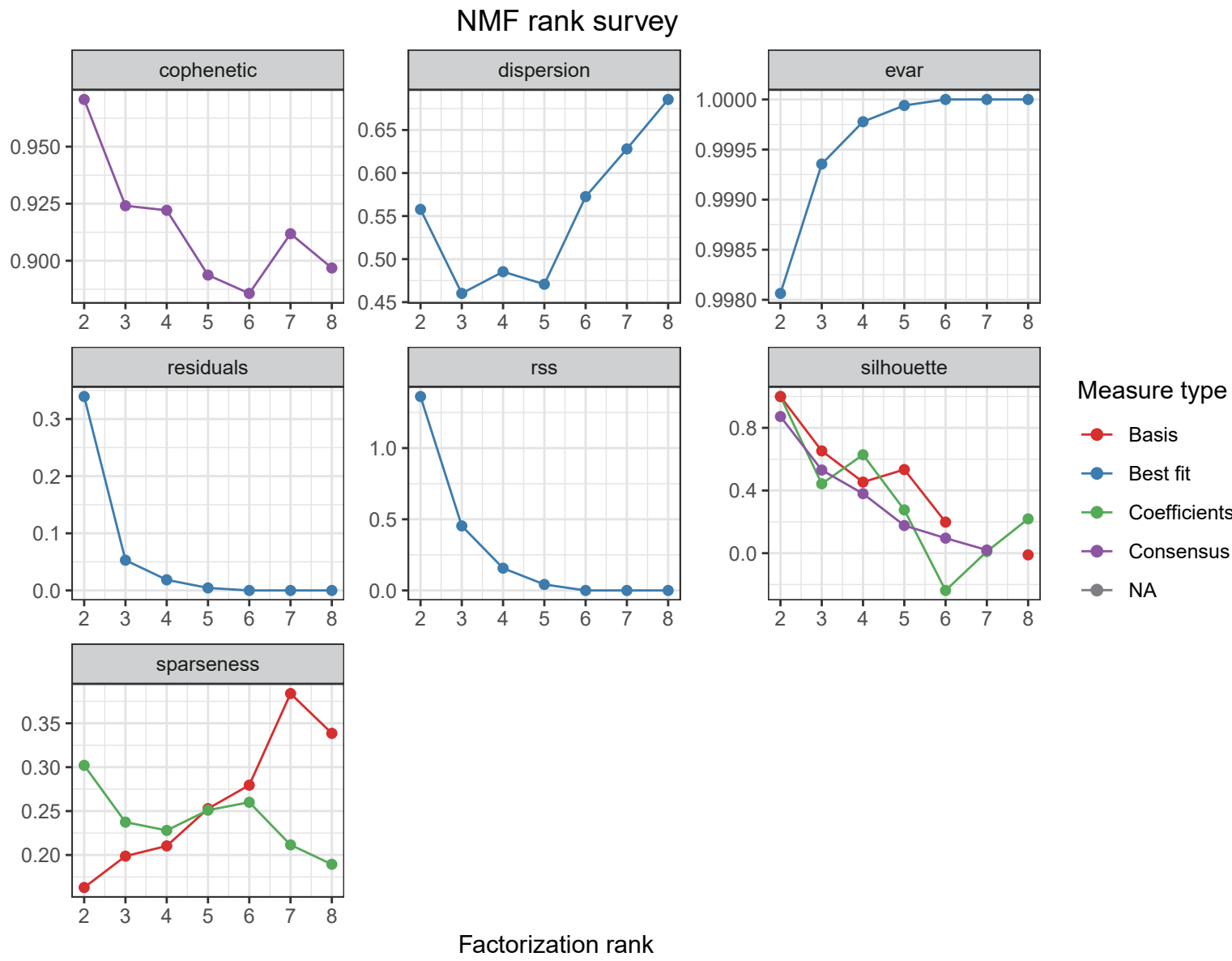

F

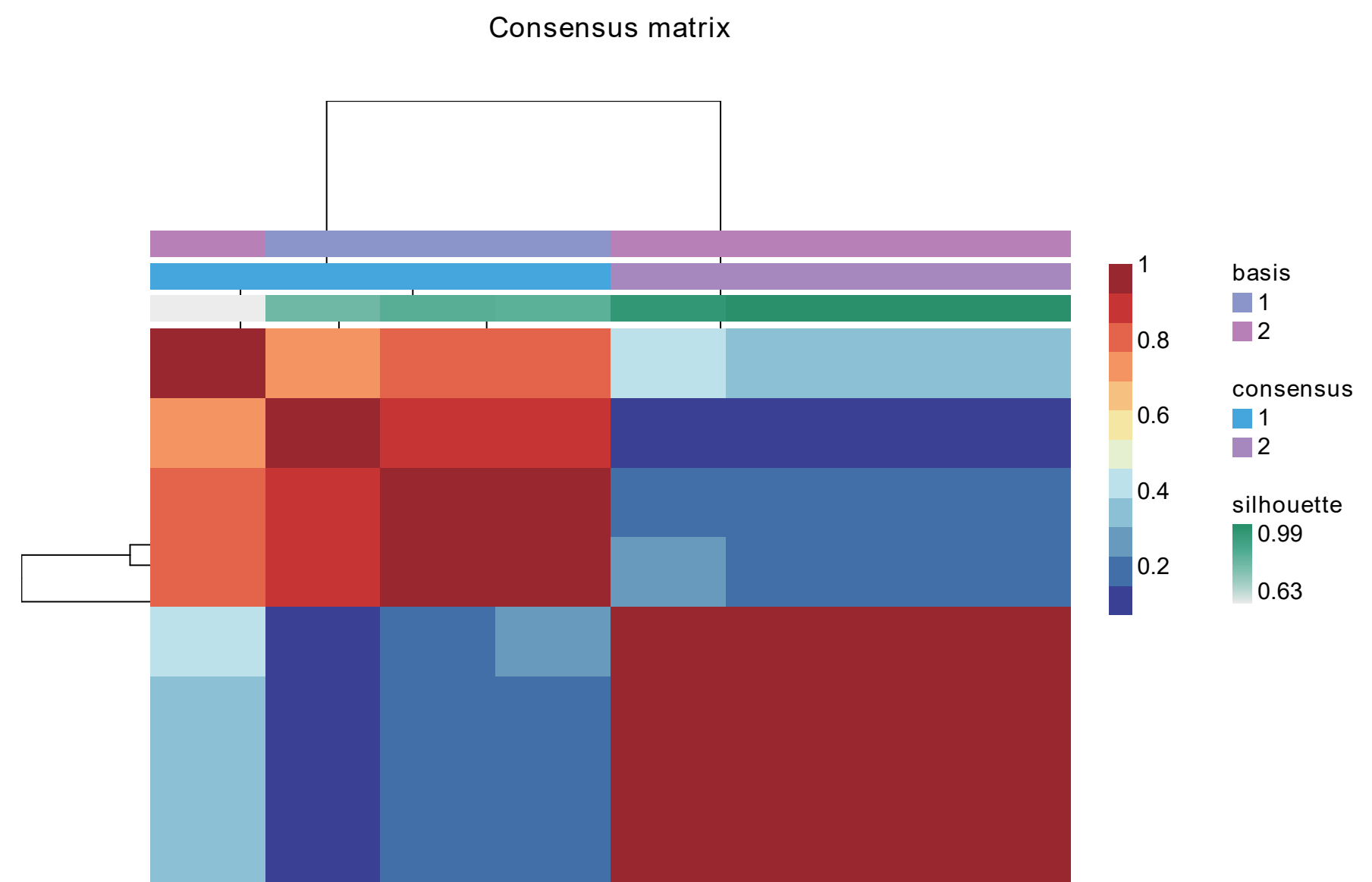

Supplement: S4 Fig — A, C, and E are the classification index plots of the sample prediction for GSE 28829, GSE 41571, and GSE120521 based on significantly different m6A regulators, respectively. B, D, and F are the clustering results of NMF on the GSE 28829, GSE 41571, and GSE 120521 datasets, respectively. (PDF) [file pone.0336139.s004.pdf]

A

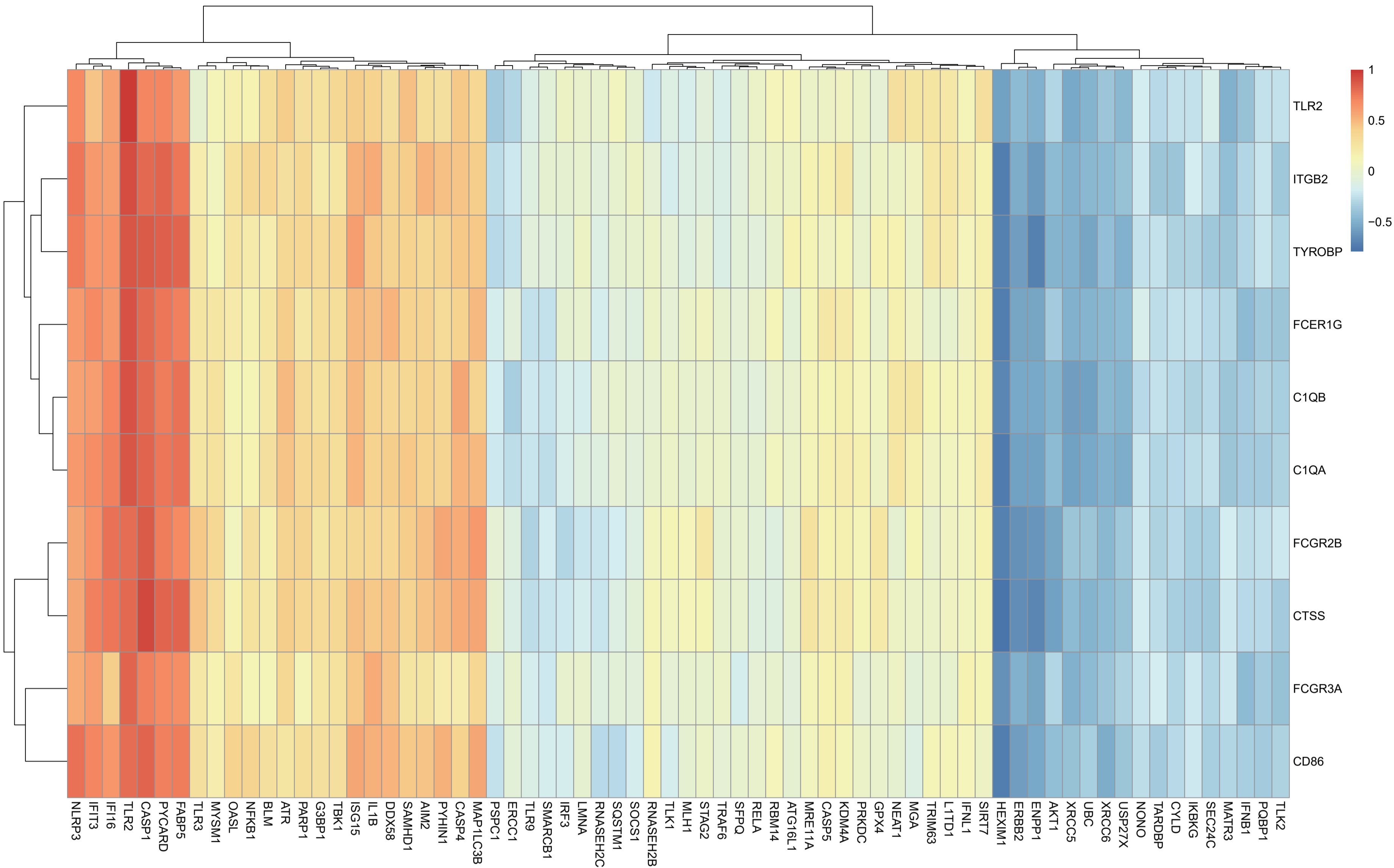

B

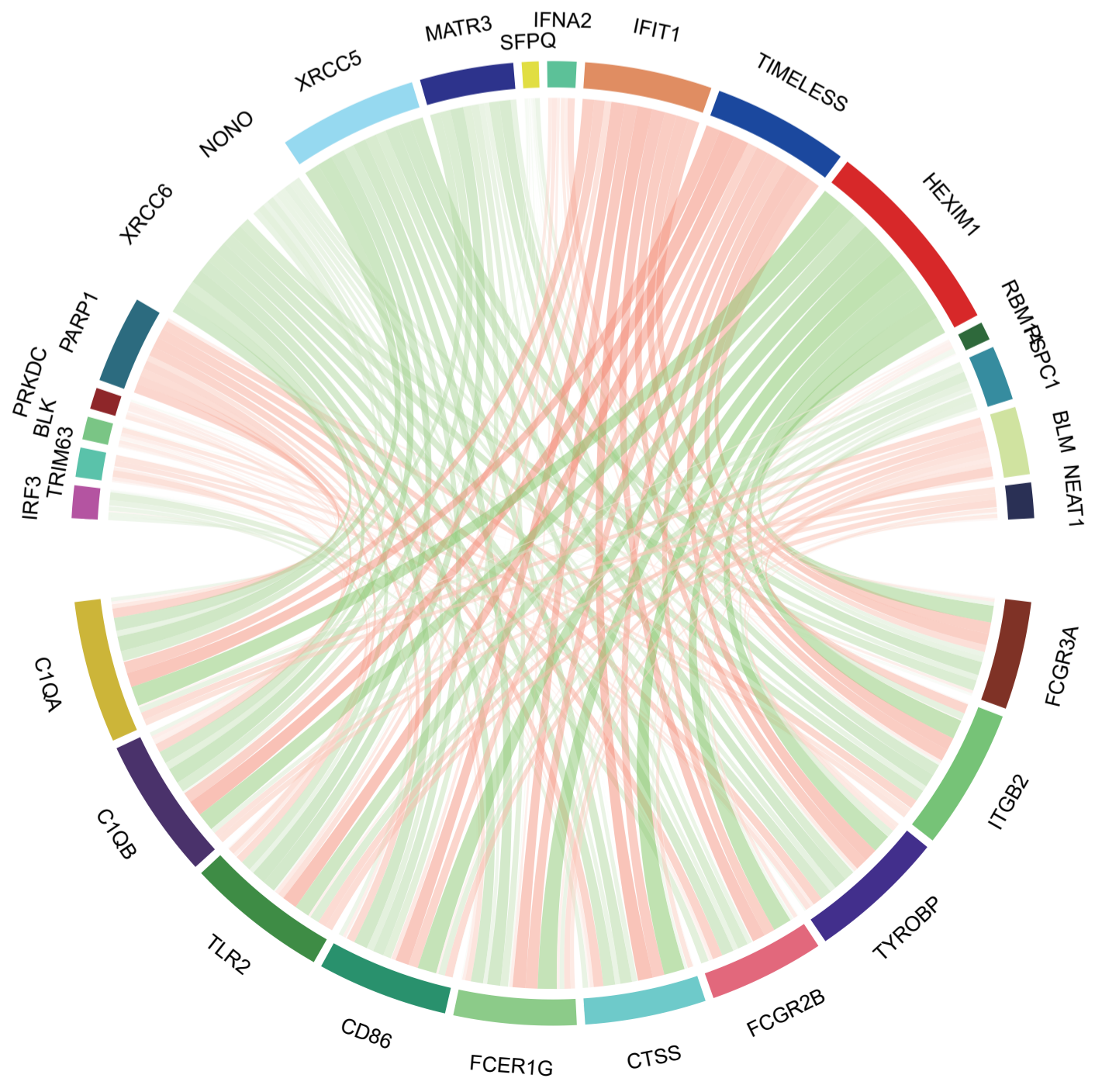

C

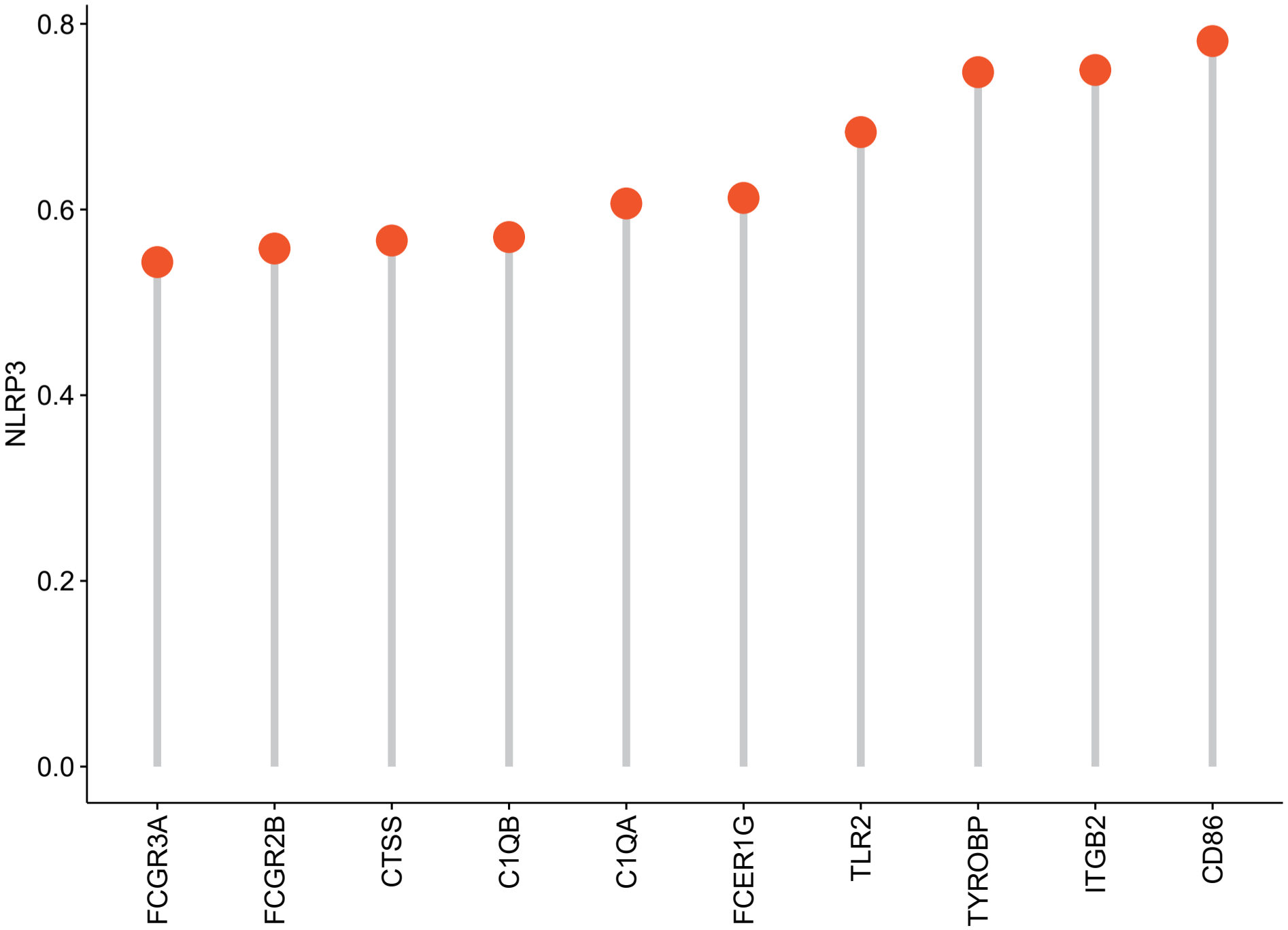

Supplement: S5 Fig — A. Heatmap of the correlation between Hub genes and genes associated with cGAS-STING. Red represents a positive correlation, and blue represents a negative correlation. B. Circular plot of the correlation of Hub genes and genes associated with STING-IRF3. The wired color between genes in the figure represents the degree of correlation, red represents the positive correlation, green represents the negative correlation, and color depth represents the correlation size. C. Lollipop plot of correlation between Hub gene and STING-NLRP3 related genes. The abscissa represents the hub gene, the ordinate represents the correlation with the NLRP3 gene, and the master height represents the correlation size. (PDF) [file pone.0336139.s005.pdf]
