# Supplementary material for: Identification and validation of diagnostic markers of atherosclerosis progression via bioinformatics strategies
Source: PLoS One. 2025 Dec 5;20(12):e0336139. doi: 10.1371/journal.pone.0336139 (PMC12680236; doi:10.1371/journal.pone.0336139)
Supplement: S1 Text — (DOCX) [file pone.0336139.s006.docx]

**Relationship between hub genes and m6A regulators, STING pathways**

**Supplementary methods**

**1.1 m6A regulator analysis**

We first calculated the Pearson correlation and interaction relationships between the hub and m6A genes. Then, from the Yongsheng Li et al. information on the m6A regulators was obtained, including 11 readers, 7 writers, and 2 erasers. We examined the differential expression of m6A regulators in atherosclerosis data and Pearson's correlation[1] .Finally, we used the R package NMF (v0.23.0) to explore the role of m6A regulators in atherosclerosis[2].

**1.2 Correlation analysis of DEGs associated with cellular ER stress and mitochondrial damage and STING pathways**

We also investigate the relationship between DEGs related to ER stress, mitochondrial damage, and STING pathways, including cGAS-STING, STING-IRF3, STING-NLRP3 pathways. Correlation heat maps, ring maps, and lollipop maps were generated using the R packages pheatmap (v1.0.12), circlize (v0.4.13), and ggpubr (v0.4.0), respectively[3].

**2. Results**

**Supplement 2.1 Relationship between hub genes and m6A regulators**

It has been shown that m6A modification is associated with atherosclerosis, and to further explore the relationship of hub gene and m6A, we calculated the correlation of hub gene and m6A gene. Results (S1A Fig) showed that m6A gene was positively associated with hub gene, with the highest correlation with CTSS expression and the lowest correlation with TLR2 expression. S2B Fig shows a significant interaction between the Pearson correlation of the m6A gene and the hub genes.

To further analyze the relationship between m6A and atherosclerosis, we checked the expression of m6A regulators in the early and advanced stages. S2A Fig shows the heat map of 19 regulators of readers, writers, and erasers in the early and late atherosclerosis. Overall, the expression of writers and erasers is slightly higher in the early stage, but the trend is not obvious. The difference between regulators is huge; readers is not obvious in the early and advanced stages. S2B Fig analyzed the differences of each m6A regulator in the early and late stage, found that the expression of *FTO* (erasers), *METTL 14* (writers), *YTHDF 3* (readers) and *ZC 3H13* (writers) was significantly higher than in the advanced stage; the expression of *IGF2BP 2* (readers) and *IGF2BP3* (readers) was significantly higher than in the early stage. S2C Fig shows the location of the regulators on the chromosome.

Next, we further analyzed the correlation between readers, writers, and erasers at expression level. S3 Fig shows the expression correlation of m6A regulators in the dataset GSE28829, and the two validation sets GSE41571 and GSE120521, respectively. The results showed that only two expression correlations of readers (*GIFBP2, IGHBP3*) and erasers FTO showed a positive correlation in all three datasets.

Thereafter, we performed molecular typing of atherosclerosis data based on m6A regulators. The NMF method of the cophenetic diagram (S4A, C, E Fig) was the best number of clusters. Results (S4A-B Fig) samples showed atherosclerosis data GSE28829 could be divided into two categories. The same clustering operation was performed with the two validation sets, GSE41571 and GSE120521, with the same result (S4 C-F Fig).

**2.2 Hub genes correlation with the cGAS-STING, STING-IRF3, and STING-NLRP3 genes**

Since STING pathways have been proved to be linked with ER stress, mitochondrial damage, and inflammation, we paid special attention to the expression relationship of hub genes and cGAS-STING, STING-IRF3, STING-NLRP3-related genes. We calculated the correlation size of hub genes with these genes and visualized them (S5 Fig). As shown in Figure, the hub gene were highly positively correlated with genes in cGAS-STING pathway, including NLRP3, IFIT3, IFI16, CASP1, PYCARD, and FABP (S5A Fig). HEXIM1 in STING-IRF3 pathway also showed extremely high correlation with all hub genes. All of these have a positive correlation (S5B Fig). The NLRP3 in the STING-NLRP3 pathway were all positively correlated with the hub gene, and showded the highest correlation with CD 86 (S5C Fig).

**Figure Legends**

**S1 Fig. Correlation analysis between the m6A gene and the hub gene. A.** Heatmap of Pearson correlation between expression levels of Hub gene and m6A genes. **B.** Significant interaction network map of Pearson correlation for expression levels of Hub genes and m6A genes. Red dots represent the m 6A gene, and green dots represent the hub gene. Point size represents the connectivity, and line thickness represents the Pearson correlation size.

**S2 Fig. m6A regulator expression and localization.** **A.** Heatmap of the m6A regulon expression in early and late atherosclerosis. **B.** Box plot of the expression of the m6A regulon in early and late atherosclerosis. **C.** Information on the position of the m6A regulators on the chromosome.

**S3 Fig. Pearson correlation for the expression of m 6 A regulators.** **A-C** are the Pearson correlation coefficients of the significantly different m6A regulators in the GSE 22829 and in the validation sets GSE41571, GSE120521, respectively.

**S4 Fig. Type-typing analysis based on m 6A regulators. A, C, and E** are the classification index plots of the sample prediction for GSE 28829, GSE 41571, and GSE120521 based on significantly different m 6A regulators, respectively. **B, D, and F** are the clustering results of NMF on the GSE 28829, GSE 41571, and GSE 120521 datasets, respectively.

**S5 Fig. Correlation between hub gene expression and the expression of genes associated with cGAS-STING, STING-IRF3, and STING-NLRP3. A.** Heatmap of the correlation between Hub genes and genes associated with cGAS-STING. Red represents a positive correlation, and blue represents a negative correlation. **B.** Circular plot of the correlation of Hub genes and genes associated with STING-IRF3. The wired color between genes in the figure represents the degree of correlation, red represents the positive correlation, green represents the negative correlation, and color depth represents the correlation size. **C.** Lollipop plot of correlation between Hub gene and STING-NLRP3 related genes. The abscissa represents the hub gene, the ordinate represents the correlation with the NLRP3 gene, and the master height represents the correlation size.

**Reference**

1. Li, Y., et al., *Molecular characterization and clinical relevance of m6A regulators across 33 cancer types.* Molecular Cancer, 2019. **18**(1): p. 137.

2. Guan, N., et al., *Truncated Cauchy Non-Negative Matrix Factorization.* IEEE Transactions On Pattern Analysis and Machine Intelligence, 2019. **41**(1): p. 246-259.

3. Kolde, R. and M.R.J.R.p. Kolde, *Package ‘pheatmap’.* 2015. **1**(7): p. 790.
